# Effects of overexpression of *STB5* in *Saccharomyces cerevisiae* on fatty acid biosynthesis, physiology and transcriptome

**DOI:** 10.1093/femsyr/foz027

**Published:** 2019-03-29

**Authors:** Alexandra Bergman, Dóra Vitay, John Hellgren, Yun Chen, Jens Nielsen, Verena Siewers

**Affiliations:** 1 Department of Biology and Biological Engineering, Systems and Synthetic Biology, Chalmers University of Technology, Kemivägen 10, SE41296, Gothenburg, Sweden; 2 Novo Nordisk Foundation Center for Biosustainability, Chalmers University of Technology, Kemivägen 10, SE41296 Gothenburg, Sweden; 3 Novo Nordisk Foundation Center for Biosustainability, Technical University of Denmark, Kemitorvet, Building 220, DK2800 Kgs. Lyngby, Denmark

**Keywords:** *Saccharomyces cerevisiae*, *STB5*, transcription factor, NADPH, fatty acids, metabolic engineering

## Abstract

Microbial conversion of biomass to fatty acids (FA) and products derived thereof is an attractive alternative to the traditional oleochemical production route from animal and plant lipids. This study examined if NADPH-costly FA biosynthesis could be enhanced by overexpressing the transcription factor Stb5 in *Saccharomyces cerevisiae*. Stb5 activates expression of multiple genes encoding enzymes within the pentose phosphate pathway (PPP) and other NADPH-producing reactions. Overexpression of *STB5* led to a decreased growth rate and an increased free fatty acid (FFA) production during growth on glucose. The improved FFA synthetic ability in the glucose phase was shown to be independent of flux through the oxidative PPP. RNAseq analysis revealed that *STB5* overexpression had wide-ranging effects on the transcriptome in the batch phase, and appeared to cause a counterintuitive phenotype with reduced flux through the oxidative PPP. During glucose limitation, when an increased NADPH supply is likely less harmful, an overall induction of the proposed target genes of Stb5 (eg. *GND1/2*, *TAL1*, *ALD6*, *YEF1*) was observed. Taken together, the strategy of utilizing *STB5* overexpression to increase NADPH supply for reductive biosynthesis is suggested to have potential in strains engineered to have strong ability to consume excess NADPH, alleviating a potential redox imbalance.

## Teaser

*STB5 —*encoding a transcriptional activator of genes related to NADPH biosynthesis — was overexpressed in an attempt to enhance FA biosynthesis and the resulting effects were studied at different growth conditions.

## INTRODUCTION

The transformation of the current economy towards a biobased one is dependent on versatile technologies able to convert renewable feedstocks into chemical products and fuels. Within the field of metabolic engineering advances have been made to positively stimulate microbial biosynthesis of fatty acids (FA), the building blocks of oleochemicals (Pfleger, Gossing and Nielsen 2015; Marella *et al*. 2018). Oleochemicals represent a group of compounds with a diverse application range, for example in cosmetics and detergents as well as in industrial lubricants and transportation fuels. Currently, oleochemicals are predominantly produced from the FA molecules present in lipids such as vegetable oils — a global market which grew 4.5% annually the past decade due to the increased demand for biodiesel (OECD/FAO 2018). Microbial production of oleochemicals and biodiesel has distinct advantages compared to the traditional route based on plant or animal fats. A great variety of feedstocks can be utilized, including biomass with a high crop yield, hence resulting in a lower overall carbon footprint (Fargione *et al*. 2008). Furthermore, a microbe can be engineered to catalyze multiple steps of the conversion processes required to produce the sought-after product, from substrate degradation pathways to control of FA chain length and/or degree of saturation.

One of the most attractive hosts for metabolic engineering and industrial applications is the yeast *Saccharomyces cerevisiae*. Naturally, *S. cerevisiae* produces low quantities of lipids, but during the last decade it has been widely explored as a cell factory for FA and oleochemical synthesis (Zhang, Nielsen and Liu 2018). *De novo* FA synthesis is a cyclic cytosolic process, where acetyl-CoA is used as substrate to produce malonyl-CoA, which subsequently elongates FA chains by two carbons at a time, ultimately releasing fatty acyl-CoA chains. Engineering strategies to improve FA biosynthesis have generally focused on increasing the acetyl-CoA supply (de Jong *et al*. 2014; Zhou *et al*. 2016), de-regulating key-enzymes in the pathway (Shi *et al*. 2014; Ferreira *et al*. 2018a), preventing acyl-CoA mediated inhibition and/or FA degradation (Leber *et al*. 2014; Ferreira *et al*. 2018a) and overexpressing downstream pathways further converting the produced acyl-CoA or free fatty acid (FFA) (Ferreira *et al*. 2018a). FA biosynthesis consumes 2 NADPH per molecule of malonyl-CoA entering the FA elongation cycle after an initial acetyl-CoA priming reaction (Tehlivets, Scheuringer and Kohlwein 2007), resulting in a cost of 16 NADPH for one molecule of octadecanoic acid (C18). A recent study presented a rigorous reprogramming effort to turn *S. cerevisiae* into a lipogenic yeast, which partly included to re-route flux through the oxidative pentose phosphate pathway (PPP) for an increased NADPH generation (Yu *et al*. 2018). Besides the mentioned study, increasing NADPH supply remains a largely unexplored avenue to influence FA synthesis in *S. cerevisiae*.

The oxidative PPP is a major route for cytosolic NADPH generation in *S. cerevisiae*. It generates two NADPH per molecule of glucose consumed through the action of the two dehydrogenases, glucose-6-phosphate (G6P) dehydrogenase (Zwf1) and 6-phosphogluconate dehydrogenase (Gnd1/Gnd2) (Stincone *et al*. 2015). Other cytosolic routes leading to NADPH formation include the acetaldehyde dehydrogenase (Ald6), important for growth on glucose, and cytosolic isocitrate dehydrogenase (Idp2), which is essential for growth on acetate and oleate if *ZWF1* is deleted (Minard and McAlister-Henn 2005). Mitochondrial NADPH supply stems from malic enzyme (Mae1), a NAD(H) kinase (Pos5) and mitochondrial isoforms of acetaldehyde dehydrogenase (Ald4/Ald5) and isocitrate dehydrogenase (Idp1) (Miyagi, Kawai and Murata 2009). Even though the mitochondrial membrane is not directly permeable for the cofactor, mitochondrial export of citrate and uptake of oxoglutarate catalyzed by the transporter Yhm2 is suggested to cause a net export of reducing equivalents to the cytosol, demonstrated by the reduced cytosolic NADPH/NADP^+^ ratio observed in a *yhm2∆* mutant (Castegna *et al*. 2010). The NADPH/NADP^+^ ratio is believed to be more tightly regulated than the NADH/NAD^+^ ratio. For example, overexpression of cytosolic Pyc2 and NADPH-producing Mae1, either targeted to the mitochondria or the cytosol, did not generate a measurable difference in NADPH/NADP^+^ ratio while NADH/NAD^+^ levels decreased and the flux through the oxidative PPP was reduced, indicating a lower cytosolic need for NADPH (Moreira dos Santos *et al*. 2004).

As FA synthesis is carried out in the cytoplasm, the obvious native targets to increase NADPH supply are the cytosolic dehydrogenases Zwf1, Gnd1/2, Ald6 and Idp2. Of these, Ald6 is a part of the native enzymatic route generating FA from glucose, and overexpression has been attempted in combination with other modifications, mainly to increase flux towards acetyl-CoA and metabolites derived thereof (Shiba *et al*. 2007; Chen *et al*. 2013; Krivoruchko *et al*. 2013; de Jong *et al*. 2014). Idp2 is mainly responsible for NADPH generation during growth on non-fermentable carbon sources and was, together with Yhm2, one of the chosen targets to improve NADPH-supply by Yu *et al*. for FFA production (Yu *et al*. 2018). Redirection of flux through the oxidative PPP should in theory have a stronger effect on NADPH generation, as two molecules of NADPH are formed per G6P entering the pathway. However, flux through the G6P node is mainly believed to be determined by the concentrations of G6P and NADPH, suggesting that an increase in Zwf1 level may not have a great influence on the flux distribution (Stephanopoulos and Vallino 1991; Gombert *et al*. 2001). NADPH inhibits Zwf1 activity in crude cell free extracts (Llobell *et al*. 1988), which indicates that the NADPH concentration or the NADPH/NADP^+^ ratio dictates the flux entering the oxidative PPP. This was recently shown to be the case in *Escherichia coli* (Christodoulou *et al*. 2018). In yeast, overexpression of *YNO1* encoding an NADPH oxidase caused a 10-fold increase in superoxide levels, while no such increase was present in a *zwf1*∆ mutant, indicating that flux through the oxidative PPP flux dynamically increases in response to NADPH demand (Rinnerthaler *et al*. 2012). If simultaneously considering the strong thermodynamic forces driving flux through glycolysis, it is apparent that a major intervention is required to efficiently redirect flux through the oxidative PPP.

Even though metabolite levels and post-transcriptional effects are likely to influence oxidative PPP activity (Wang *et al*. 2014), it has also been suggested to be regulated on a transcriptional level. The gene *STB5* encodes a transcription factor (TF) reported to regulate multidrug resistance and oxidative stress response (Larochelle *et al*. 2006; Ouyang *et al*. 2018). In response to diamide treatment, Stb5 was shown to act as transcriptional activator of multiple genes within the PPP, including *ZWF1*, *SOL3*, *GND1*, *GND2*, *RKI1*, *TAL1* and *TKL1*, while it is believed to repress the glycolytic gene *PGI1*, whose gene product competes with Zwf1 for the substrate G6P (Larochelle *et al*. 2006). In addition, *STB5* was found to positively influence the expression of several other genes related to NADPH generation, such as *ALD6*, *IDP2*, *ILV5*, *ALD4, GOR1* and *YEF1* (Larochelle *et al*. 2006).This suggests that an increased flux through the PPP as well an increased pool of NADPH potentially could be achieved simply by enhancing the activity of *STB5*. Indeed, overexpression of *STB5* from a low-copy plasmid increased NADPH levels 2.5 times in BY4727 background (Hector *et al*. 2009). Strong overexpression of *STB5* prevented strain growth on glucose, but this growth defect was partly relieved when overexpressing a NADPH-consuming butanediol dehydrogenase, indicating that *STB5* overexpression led to a redox imbalance (Cadiere, Galeote and Dequin 2010).

**Figure 1.**
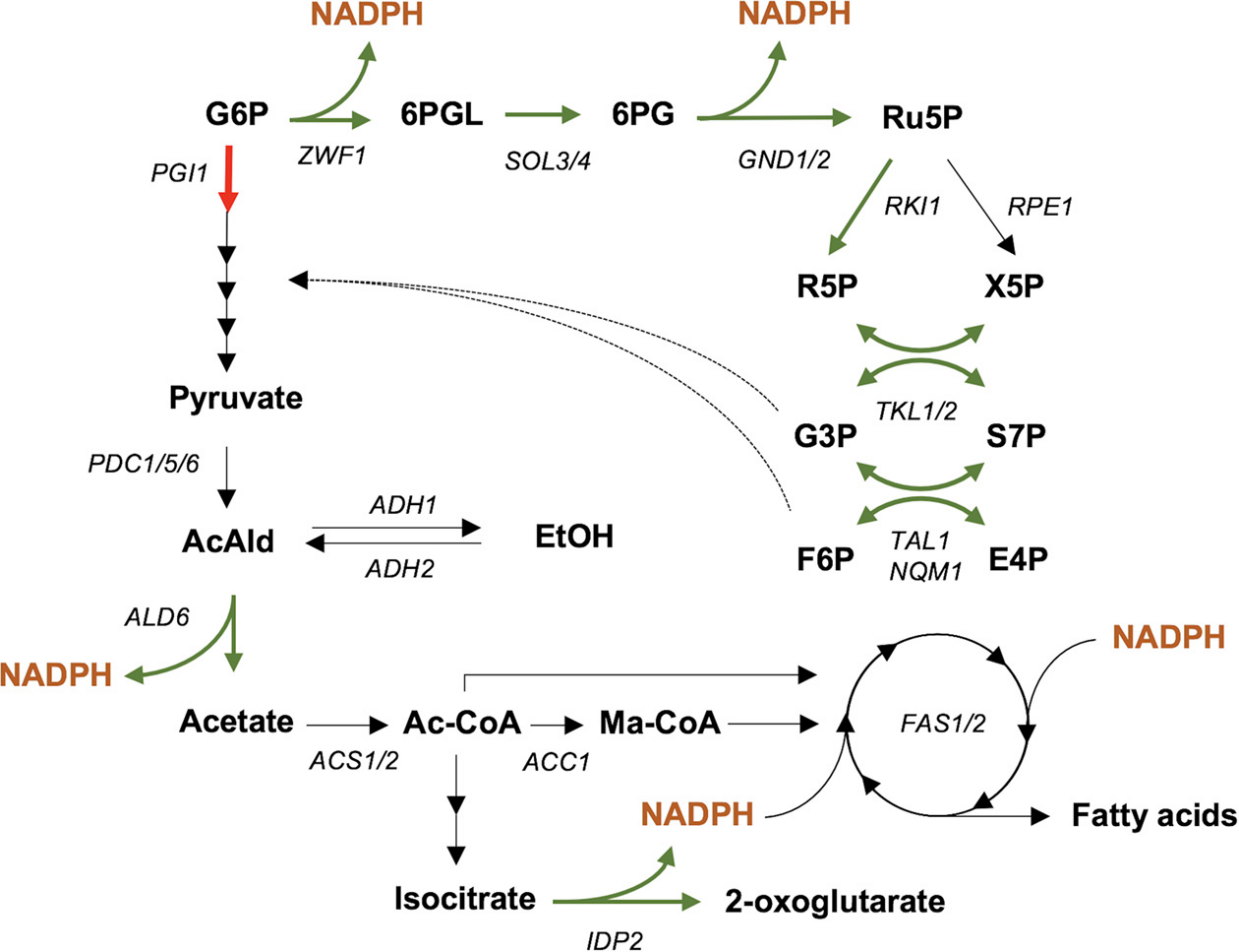
Overview of central carbon metabolism of *S. cerevisiae* and key targets of the TF Stb5. Positively and negatively regulated reactions are highlighted in green and red, respectively.

In this study, we evaluated if promoter replacement of *STB5* could be used as a tool to positively influence FA synthesis, with the hypothesis that it potentially could activate several NADPH-producing reactions simultaneously while downregulating flux through upper glycolysis. In Fig. 1, an overview of central carbon metabolism and some of the key targets of Stb5 anticipated to be perturbed are shown. If successful this strategy could replace the requirement for promoter replacement of multiple genes, e.g. fine-tuned downregulation of *PGI1* and overexpression of *ZWF1*, *GND1*, *TAL1* and *TKL1* that resulted in a 28% improvement in FA production (Yu *et al*. 2018).

## MATERIALS AND METHODS

### Strains and plasmids

Investigated yeast strains were constructed from the laboratory yeast strain CEN.PK 113–5D. Table 1 shows name, genotype and origin of all strains used and constructed under the course of the study.

**Table 1.** Strains and plasmids used and constructed within this study.

Strain	Genotype	Origin
CEN.PK 113–5D	*MAT*a *MAL2–8c SUC2 ura3–52*	P. Kötter, University of Frankfurt, Germany
AB11	*MAT*a *MAL2–8c SUC2 ura3–52 faa1∆ faa4∆*	This study
AB12	CEN.PK 113–5D P*_STB5_*Δ::P*_CUP1_*	This study
AB13	CEN.PK 113–5D P*_STB5_*Δ::P*_CYC1_*	This study
AB14	CEN.PK 113–5D P*_STB5_*Δ::P*_ADH1_*	This study
AB15	CEN.PK 113–5D P*_STB5_*Δ::P*_TPI1_*	This study
AB16	CEN.PK 113–5D P*_STB5_*Δ::P*_PGK1_*	This study
AB17	AB11 P*_STB5_*Δ::P*_CYC1_*	This study
AB18	AB11 P*_STB5_*Δ::P*_ADH1_*	This study
AB19	AB11P*_STB5_*Δ::P*_TPI1_*	This study
AB20	AB11 P*_STB5_*Δ::P*_PGK1_*	This study
AB21	AB11 *zwf1∆*	This study
AB22	AB19 *zwf1∆*	This study
AB23	AB11 *gpp1∆*	This study
AB24	AB19 *gpp1∆*	This study
AB25 (see Table 2)	AB11 pSP-GM1	This study
AB26 (see Table 2)	AB11 pAB10	This study
AB27 (see Table 2)	AB23 pSP-GM1	This study
AB28 (see Table 2)	AB23 pAB10	This study
AB29 (see Table 2)	AB24 pSP-GM1	This study
AB30 (see Table 2)	AB24 pAB10	This study
AB31	AB15 *zwf1*∆	This study

### Culture media

Strains were routinely grown in yeast extract peptone dextrose (YPD) medium or plates (10 g L^−1^ yeast extract, 20 g L^−1^ peptone and 20 g L^−1^ glucose, additional 20 g L^−1^ agar for plates), and when appropriate YPD plates containing 200 mg L^−1^ of the antibiotic G418 sulphate. When transformed with plasmids, strains were grown on SD-URA dropout plates (6.9 g L^−1^ yeast nitrogen base w/o amino acids (Formedium), 0.77 g L^−1^ complete supplement mixture w/o uracil (Formedium), 20 g L^−1^ glucose and 20 g L^−1^ agar).

Shake flask and BioLector cultivations were performed in minimal medium containing 7.5 g L^−1^ (NH_4_)_2_SO_4_, 14.4 g L^−1^ KH_2_PO_4_, 0.5 g L^−1^ MgSO_4_·7H_2_O and 20 g L^−1^ glucose, with the pH adjusted to 6.5. Sterile solutions of glucose, 1 mL L^−1^ vitamins and 2 mL L^−1^ trace metal solution (described previously (Verduyn *et al*. 1992)) were added after autoclavation. 60 mg L^−1^ uracil was added to the media if the strains did not carry a plasmid. In BioLector cultivations, a 100 mM CuSO_4_ solution was used to obtain cultivation media with final concentrations of 100, 200 or 400 µM Cu^2+^.

Bioreactor cultivations were conducted in minimal medium containing 5 g L^−1^ (NH_4_)_2_SO_4_, 3 g L^−1^ KH_2_PO_4_, 0.5 g L^−1^ MgSO_4_·7H_2_O, 20 g L^−1^ glucose, 1 mL L^−1^ vitamins, 1 mL L^−1^ trace metal solution, 60 mg L^−1^ uracil and 50 µL L^−1^ antifoam (Sigma-Aldrich).

### Strain and plasmid construction

Chromosomal modifications were introduced into CEN.PK 113–5D using a plasmid based CRISPR/Cas9 system, which required the construction of plasmids targeting each desired site of modification and promoter-replacement cassettes. Furthermore, a plasmid containing a phosphoketolase gene from *Bifidobacterium breve* and a phosphotransacetylase gene from *Clostridium kluyveri* was constructed. See a complete list of plasmids used and constructed in this study in Table 2.

**Table 2.** Description and origin of plasmids used and constructed in this study.

Plasmid	Description	Origin
pCfB2311	2µ origin; P*_SNR52_*-gRNA-T*_SUP4_*; *natMX* marker	(Stovicek *et. al*. 2015)
pCfB2312	CEN/ARS origin; P*_TEF1_*-*cas9*-T*_CYC1_*; *kanMX* marker	(Stovicek *et. al*. 2015)
pgRNA.STB5	pCfB2312 P*_SNR52_*-gRNA(*STB5*)-T*_SUP4_*	This study
pgRNA.FAA1/FAA4	pCfB2312 P*_SNR52_*-gRNA(*FAA1*)-T*_SUP4_* P*_SNR52_*-gRNA(*FAA4*)-T*_SUP4_*	This study
pgRNA.ZWF1	pCfB2312 P*_SNR52_*-gRNA(*ZWF1*)-T*_SUP4_*	This study
pgRNA.GPP1	pCfB2312 P*_SNR52_*-gRNA(*GPP1*)-T*_SUP4_*	This study
pSP-GM1	P*_TEF1_*-P*_PGK1_* bidirectional promoter (2μ, *URA3*)	(Chen *et. al*. 2012)
pAB3	pSP-GM1 P*_TEF1_*-*xfpk*(*B. breve*)	(Bergman *et. al*. 2016)
pAB10	pSP-GM1 P*_TEF1_*-*xfpk*(*B. breve*) P*_PGK1_*-*pta*(*C. kluyveri*)	This study

gRNA selections were made using the CRISPy tool (Jakounas *et al*. 2015) for gene deletions, and Deskgen's online Knockin tool (www.deskgen.com) for *STB5* promoter exchange, as it allows for guide selection within the promoter region with accompanying activity and off-target scores (Doench *et al*. 2016). The selected 20 bp guide sequences are listed in Table S1 (Supporting Information).

The plasmid CfB2311 (Stovicek *et al*. 2015) containing a gRNA cassette with the *SNR52* promoter and *SUP4* terminator, was used as an initial template for gRNA cassette amplification. The gRNA cassettes were amplified as two individual fragments, where the 20 bp targeting sequences were added as complementary overhangs on the reverse primer for the first fragment, which comprises P*_SNR52_*, and on the forward primer for the second fragment, which comprises the structural region of the gRNA and T*_SUP4_*. The resulting polymerase chain reaction (PCR) products were fused in a second PCR, generating the final gRNA cassette. All primers and oligonucleotides used in this study are listed in Table S2 (Supporting Information), and details about the gRNA-fragment assembly strategy are described in Section A. PCR were performed using Phusion high fidelity DNA polymerase (Thermo Fisher Scientific). Resulting PCR products were purified using either a Gene Jet PCR or Gel Extraction purification Kit (Thermo Fisher Scientific).

The Cas9 and gRNA were co-expressed from the centromeric plasmid pCfB2312 (Stovicek *et al*. 2015). Plasmids were constructed by linearizing the vector with Pfl23II (Thermo Fisher Scientific) and cloning either a single (P*_STB5_*, *ZWF1*, *GPP1*) or two gRNA-cassettes (*FAA1*/*FAA4*) into it by Gibson cloning (New England Biolabs). The Gibson reactions were used to transform *E. coli DH5alpha* according to the method of Inoue *et al*. (Inoue, Nojima and Okayama 1990). Transformants were used to inoculate 5 mL LB-medium with 80 mg L^−1^ ampicillin and grown at 37°C overnight. Plasmids were extracted using a GeneJET Plasmid Miniprep Kit (Thermo Fisher Scientific). The obtained plasmids were confirmed by sequencing (Eurofins Genomics), and named pgRNA.STB5, pgRNA.FAA1/FAA4, pgRNA.ZWF1 and pgRNA.GPP1.

The yeast promoters of *CUP1*, *CYC1*, *ADH1*, *TPI1* and *PGK1* were amplified from genomic DNA and flanked with 237 and 300 bp long *STB5* recognition sequences. The upstream sequence was located 662 bp away from the start codon, and the downstream recognition sequence was the 300 first base pairs of the gene (see Table S2, Section B, Supporting Information, for primer sequences and details about fragment assembly). The obtained fusion constructs were used as double stranded donors in the CRISPR/Cas9 transformations, and thus deleted 662 bp of the native promoter when integrated upstream of *STB5*.

Yeast transformations of freshly prepared competent cells were performed using the LiAc/SS carrier DNA/PEG method (Gietz and Schiestl 2007). One nmol of (each) 120 bp DS-repair oligo (Table S2, Section C, Supporting Information) and 1 µg of pgRNA-plasmid were used in transformations to achieve gene deletions. For promoter replacements, 1 µg of repair fragment (i.e. promoter constructs) and pgRNA plasmid were used. As a control, each pgRNA-plasmid was transformed without DS-oligo to verify its functionality. After a heat chock, cells were resuspended in YPD and incubated at 30°C for 2–3 h to allow expression of the *kanMX* marker gene. Transformants were selected on YPD + G418 plates, incubated for at least 2 days at 30°C, and restreaked twice before confirming the deletions and integrations with colony PCR and sequencing. The pgRNA plasmids were looped out by cultivating strains in 5 mL YPD overnight, streaking 10 µL on a YPD plate. As soon as individual colonies were formed, 10 of these were replica plated on YPD and YPD + G418 plates to find isolates that had lost the plasmid. Sequential modifications were performed according to Table 1.

Plasmid pAB10 was constructed based on plasmid pAB3 (Bergman *et al*. 2016). The phosphotransacetylase gene from *C. kluyveri* (*pta*(CK)) was codon optimized for use in *S. cerevisiae* (Genscript, see Table S3, Supporting Information, for sequence). P*_PGK1_* and T*_CYC1_* from pSP-GM1 (Chen *et al*. 2012) and *pta*(CK) were amplified using the primers listed in Table S1, Section D (Supporting Information), after which the three fragments were fused. Plasmid pAB3 was linearized using SmaI/XhoI, purified from gel, and the P*_PGK1_*-*pta*(CK)-T*_CYC1_* fusion fragment was inserted into the pAB3-backbone using homologous recombination in yeast as described previously (Bergman *et al*. 2016). The resulting plasmid pAB10 or pSP-GM1 were used to transform strains AB11, AB23 and AB24, generating strains AB25-AB30.

### Cultivation procedures

#### BioLector

Growth behavior of promoter-exchanged strains or strains carrying plasmids were evaluated in a BioLector (m2p-labs, Baesweiler, Germany). Cultures were prepared in biological triplicates. Single colonies were cultivated in 3 mL minimal medium overnight at 30°C and 200 rpm. The precultures were used to inoculate 1 mL of medium to an initial OD_600_ of 0.1 using minimal medium containing either 0, 100, 200 or 400 µM Cu^2+^. Cultivations were conducted at 30°C with 1200 rpm shaking in a 48-well microtiter plate (MTP-48-B FlowerPlate, m2p-labs). The optical density was measured with 15 min intervals until strains reached stationary phase at a filter gain of 30. Maximum specific growth rates (µ_max_) were calculated from the slope of the ln(OD_600_) plotted against time during exponential phase.

#### Shake flasks

Cultivations of strains for NADPH assay and FFA quantification were performed in biological triplicates, and strains to be evaluated in the survival assay were cultivated in biological duplicates. Single colonies were used to inoculate 3 mL of medium cultivated overnight at 30°C and 200 rpm. The precultures were used to inoculate 20 mL of medium in 100 mL unbaffled shake flasks. The initial OD_600_ was set to 0.005 to 0.1 depending on their growth rate in order to be able to synchronize time of harvest. Flasks were incubated at 30°C with 200 rpm orbital shaking. Samples were harvested around an OD_600_ 0.6–1.0 to be used in NADPH and survival assay (see sections ‘NADPH assay’ and ‘Survival assay’ for processing). For FA quantification, samples were taken at the end of exponential phase and after 72 h of cultivation (i.e. stationary phase) by withdrawing cells and media, chilling on ice while quickly measuring OD_600_ and thereafter storing the samples at -20°C until time of sample preparation (see section ‘FAME analysis’ for processing).

#### Bioreactor cultivations

Bioreactor cultivations were conducted in biological quadruplicates. Precultures were prepared in 5 mL of minimal medium in 15 mL cultivation tubes using single colonies as inoculum for each replicate, and grown overnight at 30°C and 200 rpm orbital shaking. The precultures were used to inoculate 50 mL minimal medium in 250 mL unbaffled shake flasks to an OD_600_ of 0.05–0.1 in order to scale up the preculture. The shake flasks were once again incubated at 30°C and 200 rpm, until they reached an OD_600_ of 0.8–1.0, at which a defined volume of each cell culture was centrifuged, the cell pellet was re-suspended in 50 mL fresh minimal medium for bioreactor cultivation, and used to inoculate the bioreactors (DasGip Parallel Bioreactor Systems for Microbiology (Eppendorf)) to an initial OD_600_ of 0.1 in a volume of 600 mL. During the course of the cultivation, the temperature was set to 30°C, stirring to 600 rpm and aeration to 36 L h^−1^ (0.1 VVM). The pH was adjusted to 5.0 using 2 M solutions of HCl and KOH. Composition of the exhaust gas was examined using a DasGip GA4 gas analyzer (Eppendorf). The strains were allowed to grow until early ethanol phase, after which feeding and outflow was turned on corresponding to a dilution rate of 0.1 h^−1^_._ Samples for OD_600_ and high-performance liquid chromatography (HPLC) were taken continuously during exponential growth phase and at a couple of times during chemostat to verify entry into steady state conditions. Physiological parameters and fluxes (q) for the batch phase were calculated using a cell dry weight/OD_600_ conversion factor obtained during mid exponential phase (OD_600_ ∼1.0–1.5). A biomass composition of CH_1.8_O_0.5_N_0.2_ was assumed (Villadsen, Nielsen and Lidén 2011).

Samples for RNAseq, OD_600_, cell dry weight and HPLC analysis were simultaneously taken at three times points: in mid exponential phase, early ethanol phase and in chemostat conditions. For RNAseq, cell suspension from the bioreactor was directly cooled on crushed ice and centrifuged for 5 min at 5000 g. The cell pellet was washed with ice-cold phosphate-buffered saline (PBS), transferred to Eppendorf tubes and re-centrifuged at 0°C and 10 000 g. PBS was removed and pellets were snap-frozen in liquid N_2_. Cell pellets for RNAseq analysis were stored at -80°C until being processed.

### NADPH assay

NADPH was quantified using an NADP/NADPH Quantification Kit (Sigma-Aldrich). Strains to be evaluated for NADPH content were cultivated in biological triplicates as described above. For each sample harvested, 5 mL of cell culture of an OD_600_ between 0.5–1.0 was immediately quenched in 20 mL -40°C methanol, centrifuged at 4000 g and -10°C for 3 min. The collected cell pellets were re-dissolved in 1 mL -40°C methanol, transferred to pre-cooled 1.5 mL tubes, centrifuged at 10 000 g and -10°C for 1 min, after which the supernatant was removed and the sample was snap-frozen in liquid N_2_ and thereafter stored at −80°C_._

The same day samples were to be used for NADPH quantification, the samples were freeze dried at 0.1 mbar using a Christ alpha 2–4 LSC (Christ Gefriertrocknungsanlagen) for 4 h. Thereafter, samples were dissolved in 1 mL NADP/NADPH Extraction Buffer, transferred to a pre-cooled 2 mL tubes with 0.5 mm glass beads and homogenized using a FastPrep -24 (MP Biomedicals) (4 cycles of 5.5 m/s for 30 s, 5 min resting on ice in between runs). The homogenized mixtures were transferred to pre-cooled 1.5 mL tubes, centrifuged at maximal speed in a table top centrifuge for 5 min at 0°C, and the supernatant was collected and kept on ice. The majority of the volume of the collected samples was run through 10 kDa cut-off spin filters to deprotonize the samples and the filtrates were used to quantify the NADPH according to the manufacturer's protocol. The remaining crude cell free extract was used for protein concentration determination with a Pierce BCA Protein Assay Kit (Thermo Fisher Scientific) according to the manufacturer's instructions. NADPH readings were normalized to protein content.

### Survival assay

Strains to be evaluated for survival in response to H_2_O_2_ were cultivated in biological duplicates as described above. For each sample harvested, 4 mL of cell culture was immediately added to 4 mL of freshly prepared minimal medium containing H_2_O_2_ at concentrations of 0, 2, 4 and 8 mM, resulting in active hydrogen peroxide concentrations of 0, 1, 2 and 4 mM. The tubes were incubated at 30°C and 200 rpm shaking for 30 min, after which the tubes were centrifuged at 4000 g for 5 min. Cells were washed in 10 mL deionized H_2_O, recentrifuged and once again resuspended in 10 mL deionized H_2_O. The OD_600_ was measured in duplicates, dilutions of each sample were made and 10 µL corresponding to approximately 50 cells (based on the assumption that 1 mL of cell suspension of OD_600_ of 1.0 contains 3 × 10^7^ cells) were spotted in duplicates for each sample. The average colony counts were calculated based on two biological (shake flasks) and technical (plate streaks) replicates (n = 4).

### Extracellular metabolite analysis

In order to quantify extracellular metabolites, fermentation samples were filtered through a 0.45 μm nylon filter (VWR International AB) and analysed with HPLC. The HPLC system UltiMate® 3000 (Dionex) was utilized equipped with an Aminex® HPX-87H ion exclusion column (Bio-Rad). The system was operated using 5 mM H_2_SO_4_ as eluent at a flow rate of 0.6 mL/min and a column temperature of 45°C. Quantification of glucose, glycerol, acetate and ethanol was performed using a refractive index detector (512 μRIU), while pyruvate and succinate were quantified using an UV detector.

### FAME analysis

FFA produced by strains with mutations *faa1∆ faa4∆* were simultaneously extracted and derivatized to methyl esters using a protocol modified from Haushalter et al (Haushalter *et al*. 2014). For each sample, 250 µL of cell culture (cells + medium) was transferred to a glass vial; 15 µL of tetrabutylammonium hydroxide was added, immediately followed by 250 µL of 200 mM methyl iodide and 100 mg L^−1^ pentadecanoic acid (internal standard) in dichloromethane (DCM). The tube was capped and samples placed in a vortex mixer for 30 min at 1400 rpm. To promote phase separation, the samples were centrifuged after which 150 µL of the DCM layer was transferred to a GC vial with glass insert. The samples were evaporated for 30 min using a miVac sample concentrator (SP Scientific), and as a last step resuspended in 150 µL hexane. Using a gas chromatograph (Focus GC, Thermo Fisher Scientific) equipped with a Zebron ZB-5MS GUARDIAN capillary column (30 m × 0.25 mm × 0.25 μm, Phenomenex) and a Flame Ionization Detector (FID, Thermo Fisher Scientific), the derivatized FAME samples were analyzed. The injection volume was 1 µL and the inlet temperature was set to 280°C. The GC program was as follows: initial temperature 50°C; hold for 2 min; ramp to 140°C during 3 min; ramp to 280°C during 14 min; hold for 3 min. The flow rate of the carrier gas (helium) was 1.0 mL min^−1^. The produced FAMEs were quantified by the co-analysis of FAME standards of physiologically relevant chain length (C8-C18) and pentadecanoic acid (C15). The obtained data were processed with Xcalibur software.

### RNAseq sample preparation, profiling and analysis

Cell pellets were thawed on ice, and RNA was extracted using an RNeasy Mini Kit (Qiagen). Samples were treated with TURBO DNase (Thermo Fischer Scientific) to remove genomic DNA contamination. RNA integrity was evaluated with a 2100 Bioanalyzer (Agilent Technologies). RNA samples were processed with a TruSeq Stranded mRNA HT Sample Prep Kit (Illumina) to generate a poly-A enriched cDNA library, and samples were sequenced using a NextSeq 500 (Illumina), paired end, 2 × 76 bp. Read pairs ranged between 3.0 to 4.1 million. The raw data can be retrieved from the European Nucleotide Archive with access number ERP112694. The raw reads were processed with the NGI RNAseq Pipeline (https://github.com/SciLifeLab/NGI-RNAseq) version 1.4, see Table S4 (Supporting Information) for more information. The S288c reference genome was used to map the reads (https://www.ensembl.org/Saccharomyces_cerevisiae/Info/Index), and the percentage of uniquely mapped reads was between 86.5–93.6%. Raw read counts were calculated with feature Counts (Liao, Smyth and Shi 2014) and FPKM values calculated with StringTie (Pertea *et al*. 2015). Differential gene expression analysis was performed with DESeq2 (Love, Huber and Anders 2014), adjusted *P*-values were calculated according to the method of Benjamini–Hochberg. The Platform for Integrative Analysis of Omics (PIANO) R package (Varemo, Nielsen and Nookaew 2013) was utilized to analyse enriched GO terms and perform reporter TF analysis, where the adjusted *P*-values and log_2_ fold change from the differential gene expression analysis were used as input, except for the GSA methods maxmean, gsea and page, which used Wald statistics from DESeq2 as input. GO term annotations were collected from Ensembl (www.ensembl.org), and the interaction between TFs and genes, based on both binding and expression evidence, from Yeastract (http://www.yeastract.com). From the reporter TF analysis, the top five scoring TFs from the distinct directional up and distinct directional down regulation for each condition were selected and reported with their significance calculated from a null-distribution and adjusted with the Benjamini–Hochberg method.

## RESULTS

### Overexpression of *STB5* reduces maximum specific growth rate and final biomass concentration on glucose

As a previous study had reported that strong overexpression of *STB5* disabled a wine yeast derivative to grow on glucose (Cadiere, Galeote and Dequin 2010), we initially decided to replace the native promoter of *STB5* with the Cu^2+^-responsive promoter of *CUP1* (P*_CUP1_*) to investigate if and at what level of induction *STB5* overexpression would repress growth on glucose in CEN.PK113–5D. The maximum specific growth rates of the control and the P*_CUP1_*-*STB5* strain treated with Cu^2+^ concentrations ranging from 0–400 µM are listed in Table 3A.

**Table 3.** Maximum specific growth rates (µ(max) [h^−1^]) of *STB5*-promoter exchanged strains in CEN.PK113–5D background.

***A)STB5overexpression mediated by copper induction***				
Cu^2+^ (µM)	Control (CEN.PK 113–5D)		P*_CUP1_*-*STB5* (AB12)	
**‘0’**	0.317 ± 0.034		0.344 ± 0.018	
**100**	0.254 ± 0.014		0.259 ± 0.003	
**200**	0.284 ± 0.014		0.227 ± 0.008 *	
**400**	0.181 ± 0.004		0.107 ± 0.021 *	
***B)STB5overexpression mediated by constitutive promoters***				
**Control (CEN.PK 113–5D)**	**P*_CYC1_*-*STB5* (AB13)**	**P*_ADH1_*-*STB5* (AB14)**	**P*_TPI1_*-*STB5* (AB15)**	**P*_PGK1_*-*STB5* (AB16)**
0.326 ± 0.012	0.265 ± 0.006 *	0.239 ± 0.002 *	0.246 ± 0.009 *	0.219 ± 0.014 *

Asterisks (*) indicate a significant difference (*P *< 0.05; Student´s t-test, two-sided) compared to the control at the same conditions. Strains were grown in minimal media with 2% glucose and 60 mg/L uracil. ‘Zero µM’ Cu^2+^ concentration in reality corresponds to approximately 6 µM due to the presence of Cu^2+^ in the trace metal solution added to the media. Values correspond to the average of biological triplicates ± standard deviation.

Cu^2+^ concentrations of 100, 200 and 400 µM increasingly reduced the µ_max_ of the control, which is to be expected due to the toxicity of copper (Avery, Howlett and Radice 1996). At zero and 100 µM, there was no difference in µ_max_ between the two strains, while a greater decrease was observed for P*_CUP1_*-*STB5* at 200 and 400 µM, corresponding to approximately 80 and 60% of the growth rate observed for the control at the same Cu^2+^ concentration. The maximum cell density was nevertheless exclusively reduced in P*_CUP1_*-*STB5* at all tested concentrations of Cu^2^^+^ exceeding zero, by 13–20% of what was observed for the control (Table S5, Supporting Information).

It cannot be excluded that *STB5* expression was induced to a certain extent in the control strain as well, as Cu^2+^ toxicity is believed to be mediated through the formation of reactive oxygen species (Valko, Morris and Cronin 2005), which possibly could activate a redox stress response mediated by Stb5 (Larochelle *et al*. 2006). If so, the replacement of the native *STB5* promoter with P*_CUP1_* shows that the effects of Stb5 binding can be intensified beyond the natural level of induction. Irrespective of such effects, we show that an elevated level of *STB5* expression reduces but does not entirely prevent growth on glucose in CEN.PK113–5D, as previously observed by Cadiere, Galeote and Dequin (2010).

In order to avoid the toxic effect of copper, we decided to evaluate a set of yeast constitutive promoters of varying strength. The four endogenous promoters of *CYC1*, *ADH1*, *TPI1* and *PGK1* were selected to increase *STB5* expression, where P*_CYC1_* represent a weaker promoter whereas P*_ADH1_*, P*_TPI1_* and P*_PGK1_* are considered stronger with varying relative activities (Partow *et al*. 2010; Peng *et al*. 2015). Consistent with the Cu^2+^ induction experiments, the constitutive promoter exchanges resulted in a significantly decreased µ_max_ in all constructed strains (Table 3B). Replacement of the native promoter with P*_CYC1_* reduced growth rate to approximately 80% of the control. The strongest effect was observed for the glycolytic promoter P*_PGK1_*, for which µ_max_ was close to 0.22 h^−1^, corresponding to 67% of the control. Similar to when utilizing the inducible P*_CUP1_* promoter, final biomass concentrations were also reduced in the constitutive *STB5* overexpressing strains, ranging from 13% for P*_CYC1_* to 21–22% for P*_ADH1_*, P*_TPI1_* and P*_PGK1_* compared to the control (Table S5, Supporting Information).

### Overexpression of *STB5* fails to generate a measurable surplus of NADPH and reduces survival after H_2_O_2_ shock

After establishing that overexpression of *STB5* did not prevent growth on glucose, we wanted to investigate if its overexpression generated an increase in NADPH in CEN.PK113–5D. A previous report, which investigated the function of the *STB5* regulated gene YMR315W, showed that *STB5* overexpression from a plasmid led to a 2.5-fold increase in NADPH levels in a BY4727 background. Thus, we quantified the NADPH content of the control CEN.PK113–5D and the *STB5* promoter exchanged strains (AB13-AB16) in exponentially growing cells, but failed to measure a significant difference in NADPH levels (Figure S1, Supporting Information).

**Figure 2.**
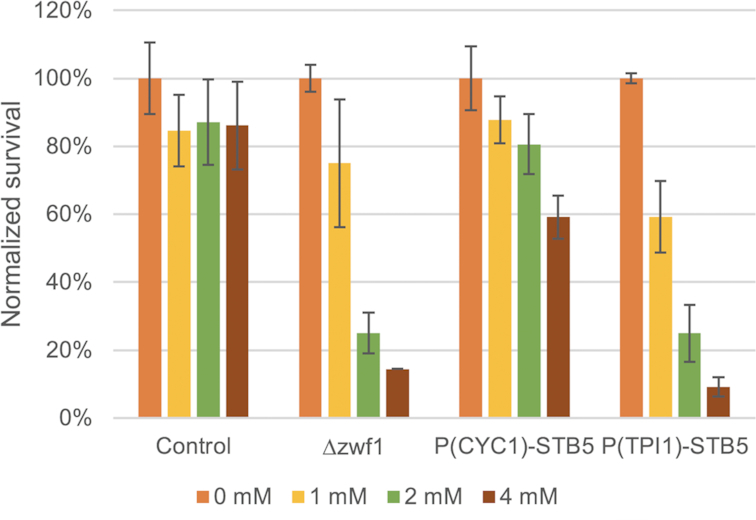
Survival of the strains CEN.PK 113–5D, *zwf1∆* and P*_CYC1_*-*STB5* and P*_TPI1_*-*STB5* after liquid H_2_O_2_-shock. The values indicate the proportion of surviving cells at each concentration compared to the value observed for each strain at 0 mM H_2_O_2_. Values are averages of two biological and two technical replicates; error bars represent ± standard deviation.

Instead, we turned to explore if the promoter exchanged strains would be more suited to handle treatment with an oxidizing agent, as NADPH supplies the reducing potential of most cellular antioxidant systems, including the glutathione system (Morano, Grant and Moye-Rowley 2012). In order to circumvent a beneficial effect of *STB5* upregulation in the wild type, which likely would occur if strains were plated on oxidative media, we instead decided to shock exponentially growing cells with different concentrations of H_2_O_2_. The experiment included the control (CEN.PK113–5D), the negative control *zwf1∆* (AB31), which is not able to catalyze the first reaction in the oxidative PPP, and two *STB5*-promoter exchanged strains: P*_CYC1_*-S*TB5* (AB13) and P*_TPI1_*-*STB5* (AB15). The proportion of H_2_O_2_-shocked cells that survived the treatment are presented in Fig. 2.

The control only showed a slightly reduced viability after H_2_O_2_ shock at all tested concentrations (1, 2 and 4 mM), indicating an efficient oxidative stress response. As expected, the *zwf1*∆ mutant showed a sharp decrease in survival at higher concentration of H_2_O_2_. The majority of the colonies were also small indicating that they indeed had been severely damaged by the exposure. Contrary to the hypothesis that overexpression of *STB5* could boost oxidative stress resistance via increased NADPH levels, survival of P*_CYC1_*-*STB5* was significantly reduced at 4 mM H_2_O_2_ compared to the control. Similarly, survival of the P*_TPI1_*-*STB5* strain was reduced to levels close to that observed for the *zwf1*∆ mutant. This shows that overexpression of *STB5* increases sensitivity to oxidative stress, possibly through a mechanism which limits flux through the oxidative PPP.

### Overexpression of *STB5* increases FA production in glucose phase in an oxidative PPP independent manner

Even though we were unable to directly demonstrate that *STB5* overexpression was linked to an increased NADPH supply, we still decided to investigate the initial question of this study: Can *STB5* overexpression be utilized as a strategy to improve NADPH dependent FA synthesis? In order to have a more efficient FA production (and drain of NADPH) the promoter replacements were integrated into the double deletion strain CEN.PK 113–5D *faa1∆faa4∆*. This strain cannot activate FFA to acyl-CoA and likely experiences less acyl-CoA mediated inhibition of FA synthesis (Faergeman and Knudsen 1997). It also excretes visual levels of FFAs into the cultivation medium which can easily be quantified (Scharnewski *et al*. 2008; Leber *et al*. 2014). Interestingly, while in *faa1∆faa4∆* strains with a strong promoter controlling *STB5*, the growth rate was reduced in a similar manner as in CEN.PK113–5D (62–72% of that observed for the control), the growth rate of P*_CYC1_*-*STB5* was not significantly altered compared to the control (Table S6, Supporting Information).

**Figure 3.**
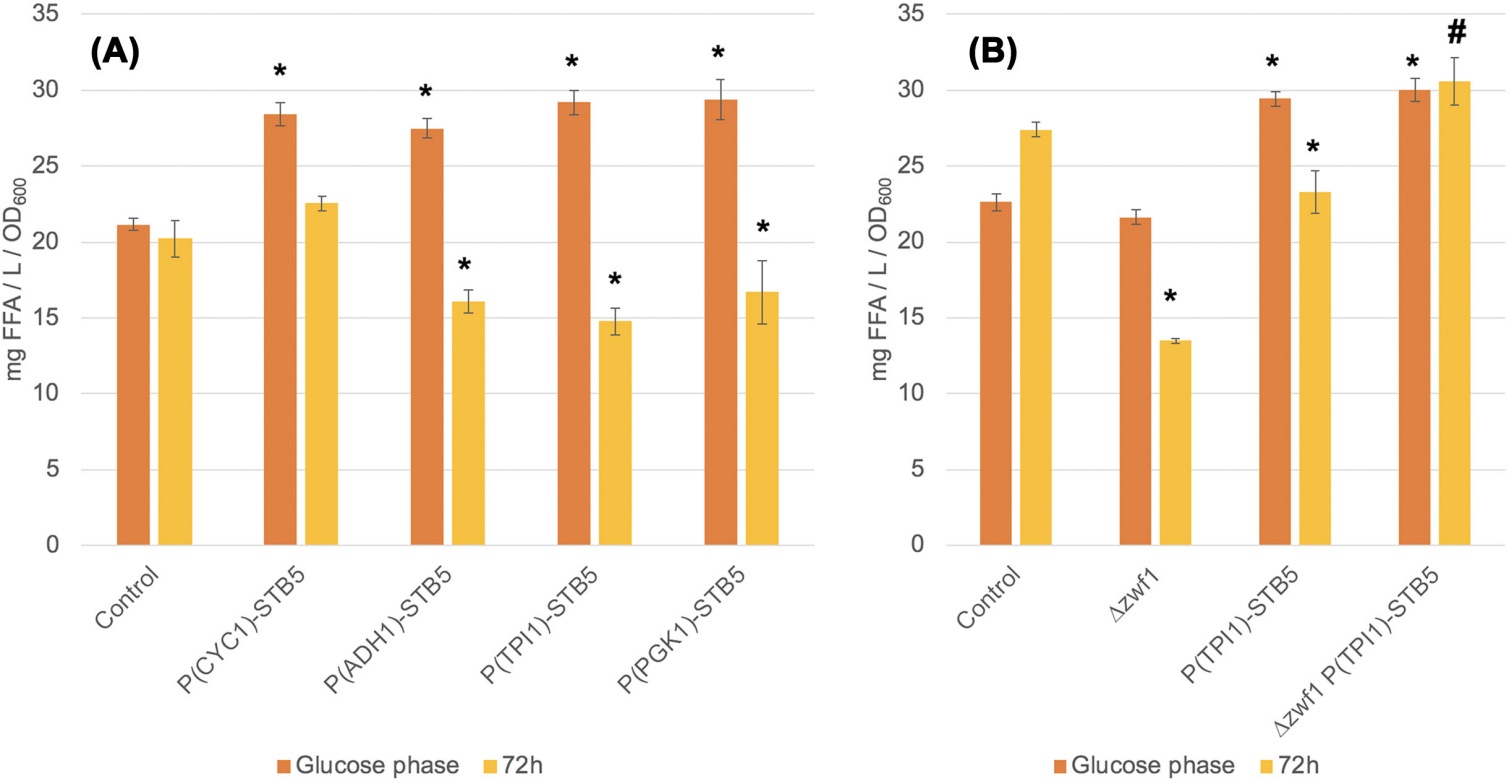
FAA quantification of CEN.PK 113–5D *faa1∆faa4∆* strains after glucose was depleted (Glucose phase) and at stationary phase (72 h). A) Influence of different levels of *STB5* overexpression on FA synthetic ability. B) Influence of *ZWF1* deletion on FA synthetic ability in the control and P*_TPI1_*-*STB5* strain (# indicates that the strain failed to consume the produced ethanol). Strains were grown in minimal medium with 2% glucose with the addition of 60 mg/L uracil. Values are averages of three biological replicates, error bars ± standard deviation. Asterisks (*) indicate a significant change from the control (*P *< 0.05; Student´s t-test, two-sided) at the corresponding sampling point.

Figure 3A shows the results of the FFA quantification of *STB5*-overexpression strains (AB17-AB20) compared with the control (AB11). The titer values (mg FFA/L) were normalized to the OD_600_ at sampling to minimize influence of sampling time point and/or biomass yield. Samples were taken at the end of the glucose phase when cells had stopped growing exponentially as well as at 72 h when cells had entered stationary phase. The quantification showed that all levels of overexpression of *STB5* had a significant beneficial effect on FFA formation in the glucose phase, resulting in a level between 130–139% of that observed for the control, while no significant difference was observed between the *STB5* overexpression strains. At 72 h, there was a small albeit non-significant increase in final FFA titers observed for the weaker promoter exchange (P*_CYC1_*), while all of the stronger promoter exchanges significantly reduced the production of FFAs to 73–83% of that observed for the control. This suggests that strong overexpression of *STB5* specifically has a negative effect on FFA synthesis in the ethanol phase.

We wondered if the specific increase in FFA synthesis during the glucose phase could be linked to an increased redirection of carbon flux through the oxidative PPP, as indicated by the regulatory targets reported previously (Larochelle *et al*. 2006). Thus, we deleted *ZWF1* in strain AB11 and AB19 and quantified the produced FFAs in the control (AB11), *zwf1∆* (AB21), P*_TPI1_*-*STB5* (AB19) and *zwf1∆* P*_TPI1_*-*STB5* (AB22) at the end of the glucose phase and at 72 h, the results of which are shown in Fig. 3B. Compared to the first experiment, FFA production in the ethanol phase was higher for the control and P*_TPI1_*-*STB5*. However, the observed trends between the control and P*_TPI1_*-*STB5* were similar to those previously observed. Interestingly, the FFA production of *zwf1∆* was not affected in the glucose phase, while a substantial decrease was observed at stationary phase, where FFA production was measured to be merely 50% of that observed for the control. Neither did the deletion of *ZWF1* influence FFA synthesis of the P*_TPI1_*-*STB5* strain in the glucose phase, suggesting that the beneficial effect of *STB5* overexpression on FFA production was independent of oxidative PPP flux. The *zwf1∆* P*_TPI1_*-*STB5* strain failed to resume growth after glucose was depleted, which is why the FFA production remained at the same level for the glucose phase and at 72 h, i.e. the presumed negative influence of *STB5* overexpression on FFA synthesis during growth on ethanol was masked.

### Synergistic effects of an increased acetyl-CoA supply and *STB5* overexpression on FA production in glucose phase

**Figure 4.**
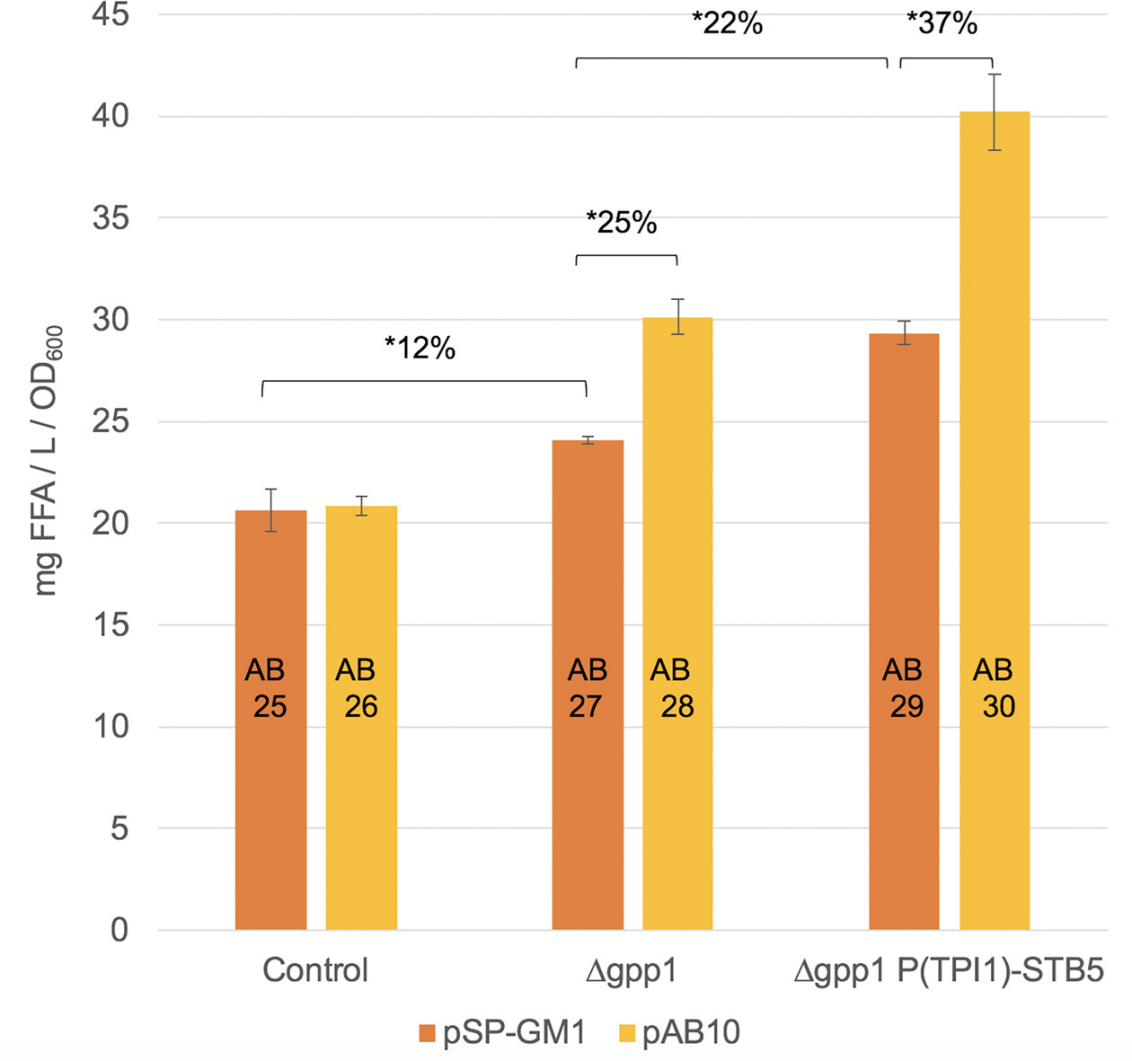
Influence of additional acetyl-CoA supply on FFA production of CEN.PK 113–5D *faa1∆faa4∆* strains on glucose. The indicated host strains were either transformed with the empty plasmid pSP-GM1 or pAB10, expressing a phosphoketolase from *B. breve* and and phosphotransacetylase from *C. kluyveri*. Strains were grown in minimal medium with 2% glucose and harvested at or close to glucose depletion. Values are averages of three biological replicates, error bars ± standard deviation. Asterisks (*) indicate a significant change (*P *< 0.05; Student´s t-test, two-sided).

Based on the observation that overexpression of *STB5* using weak (P*_CYC1_*) as well as strong promoters (P*_ADH1_*, P*_TPI1_* and P*_PGK1_*) produced a very similar beneficial effect on FA production in the glucose phase (Fig. 3A), even though the maximum specific growth rates of the respective strains differed substantially (Table S6, Supporting Information), we decided to investigate if FFA production in the glucose phase could be limited by other factors than NADPH—such as the precursor acetyl-CoA. We utilized a pathway consisting of two heterologous proteins: a phosphoketolase (Xfpk), which can convert fructose-6-phosphate or xylulose-5-phosphate to acetyl phosphate (AcP) and erythrose-4-phosphate or glyceraldehyde-3-phosphate, respectively, and a phosphotransacetylase (Pta), which catalyzes the reversible conversion of AcP to acetyl-CoA. The constructed plasmid pAB10 encodes an efficient Xfpk from *B. breve* (Bergman *et al*. 2016) and a Pta from *C. kluyveri* (Meadows *et al*. 2016). pAB10 and the empty plasmid pSP-GM1, respectively, were used to transform the strains AB11, AB23 and AB24, generating the six strains AB25-AB30. The deletion of glycerol-3-phosphatase gene *GPP1* in strain AB27-AB30 blocks the major route of endogenous hydrolysis of AcP to acetate (Bergman *et al*. 2016), which otherwise would cause energy loss, and increase CO_2_ production and respiratory demand of a strain expressing a phosphoketolase (Bergman *et al*. 2019). Fig. 4 shows the FFA titers obtained at the end of glucose phase normalized to sampling OD_600_.

The expression of the heterologous Xfpk/Pta pathway using plasmid pAB10 did not have a beneficial effect on FA production in the control strain (AB26 vs AB25). The introduction of *gpp1***∆** (AB27) had a significantly beneficial effect on FFA synthesis, which increased 12% compared the control strain (AB25). Overexpression of the Xfpk/Pta pathway in the *gpp1*∆ strain (AB28) caused a significant increase (25%) in FFA production (AB28 vs AB27), likely due to the reduced degradation of AcP into acetate. This indicates that Xfpk/Pta constitutes a functional route for increasing the acetyl-CoA supply in the *gpp1*∆ host, also shown previously (Meadows *et al*. 2016).

The *gpp1*∆ P*_TPI1_*-*STB5* strain (AB29) showed a similar level of FFA production (30 mg L^−1^ OD_600_^−1^) compared to what was observed for P*_TPI1_*-*STB5* without deletion of *GPP1* (Fig. 3A and B), and corresponded to a 22% increase compared to the *gpp1*∆ strain (AB 29 vs AB27). The introduction of the Xfpk/Pta pathway into the *gpp1*∆ P*_TPI1_*-*STB5* background (AB30) increased FFA production further, corresponding to a 37% increase compared to the same strain harboring the empty plasmid pSP-GM1 (AB30 vs AB29), and a 67% increase compared to the *gpp1*∆ strain carrying pSP-GM1 (AB30 vs AB27). These results indicate that the effects of *STB5*-overexpression and an increased acetyl-CoA supply are not just additive, but act in synergy to increase FFA synthesis.

### Overexpression of *STB5* increases expression of a majority of suggested target genes in steady state conditions while the activation is limited in batch culture

In order to obtain a better understanding for the effects resulting from *STB5* overexpression, we decided to conduct batch and continuous bioreactor cultivations of the non-modified strain (CEN.PK113–5D) and P*_TPI1_*-*STB5* (AB15). The calculated parameters maximum specific growth rate (µ_max_), biomass yield (Y_S/X_), flux-coefficients (q), carbon balance and respiratory quotient (RQ) are summarized in Table S7 (Supporting Information). As described previously, *STB5* overexpression negatively influenced the maximum specific growth rate, which caused a general decrease in the calculated specific carbon flux values in the exponential phase. If the calculated fluxes were normalized to glucose uptake rate and carbon content, glycerol, ethanol and biomass production rates still remained significantly reduced. At the same time, oxygen consumption q_O2_ increased slightly, and the RQ was reduced, indicating that the *STB5* overexpression mutant has a metabolism of more respiratory character. However, both of these parameters were altered at a non-significant level (p(q_O2_) = 0.24 and p(RQ) = 0.068).

During the course of the bioreactor cultivation, we took samples for RNAseq analysis in three different conditions: 1) in the glucose-consumption phase, when *STB5* overexpression appeared to influence strain growth the most, 2) in the ethanol-consumption phase, where a negative effect on FA synthetic ability was observed and 3) in steady state conditions—which is the preferred sampling choice for RNAseq data analysis as a difference in growth between two strains will not influence gene expression, for example as it is the case with genes related to ribosome biogenesis and stress response (Regenberg *et al*. 2006).

**Figure 5.**
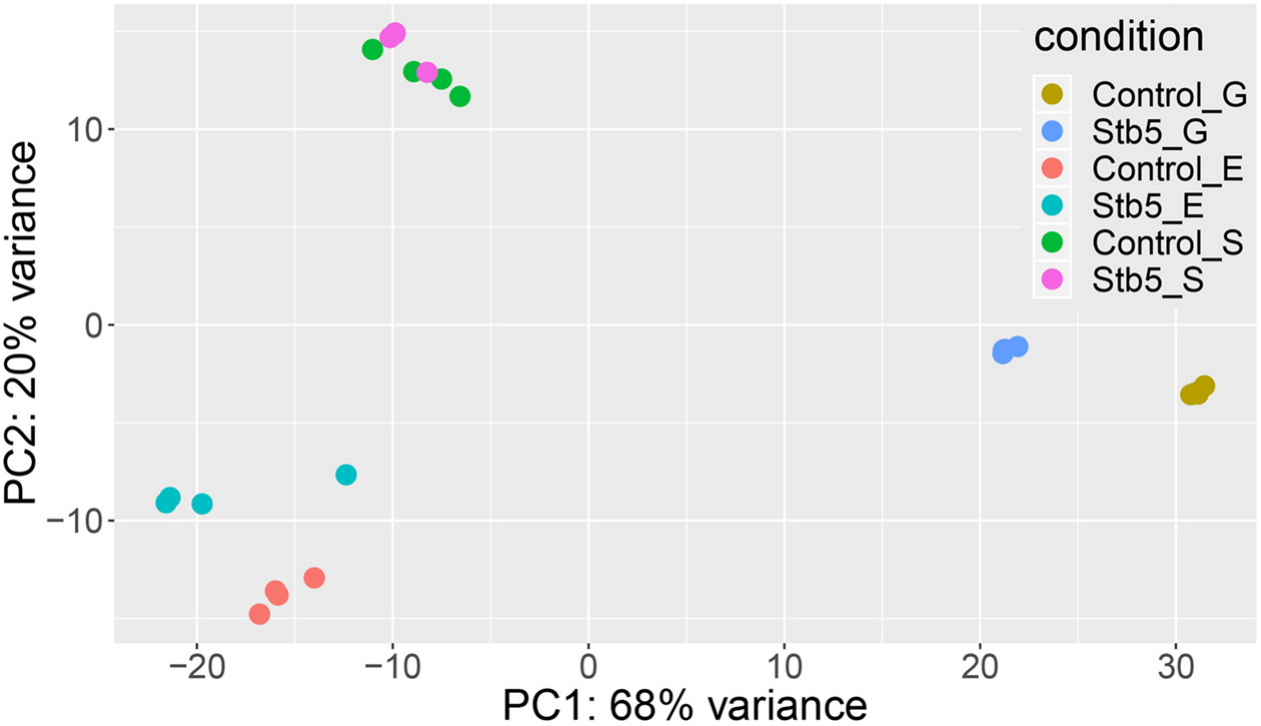
Principal component analysis plot of RNAseq data from control and *STB5* overexpression strain. ’Control’ and ‘Stb5’ indicate the control CEN.PK-113 5D and *STB5* overexpression strain AB15, respectively, while G, E and S correspond to the sampling point condition (Glucose, Ethanol and Steady state, respectively).

The overall variation of the data is visualized in a principal component analysis plot (Fig. 5), which shows a clear separation between sample groups. The largest variation was observed for samples harvested at different conditions (i.e. glucose (G), ethanol (E) and steady state (S)), consistent with the major rearrangement in metabolism associated with a shift from fermentative to respiratory growth. The largest variance within control (ref) and *STB5* overexpressing strain (s) was observed for samples harvested in the glucose phase (refG and sG for control and *STB5*-overexpression, respectively), while the variation appeared to be relatively small in steady state conditions (S). In the glucose phase, *STB5* overexpression appears to shift the cellular state to one that to a higher degree resembles respiratory conditions, indicated by the lateral shift along the first principle component in Fig. 5.

In Table 4, an overall outcome of the differential expressional analysis is shown. In the glucose phase, 3695 transcripts were significantly changed compared to the control (padj < 0.01). The number of significantly changed transcripts were lower in the ethanol phase, and decreased even further at steady state. In the glucose and ethanol phases, the majority of differentially expressed genes were positively influenced by *STB5* overexpression, as indicated by the greater number of transcripts with a large positive log_2_-fold change (LFC), while this trend could not be observed during steady state.

**Table 4.** Number of significantly changed genes and number of genes with |LFC| > 0.5 and 1.0 in P*_TPI1_*-*STB5* compared to the control strain AB15 in glucose phase, ethanol phase and steady state when cultivated in bioreactors.

	Number of transcripts				
Sampling point	padj < 0.01	LFC > 0.5	LFC > 1.0	LFC < −0.5	LFC < −1.0
Glucose phase	3695	1085	536	703	130
Ethanol phase	1568	577	266	305	82
Steady state	879	187	61	176	63

*STB5* expression was significantly enhanced compared to the control in all conditions, with a LFC of 4.83, 1.75 and 3.29 in glucose, ethanol and steady state, respectively. The normal expression levels of *STB5* were low at all conditions, with FPKM-values between 16 and 27, which for example can be compared to that of the glycolytic gene *PGK1* in the glucose phase, averaging close to 3000 (Table S8, Supporting Information).

When conducting a gene set analysis, numerous GO terms that were indicated to be distinctly downregulated upon *STB5* overexpression in the glucose phase were connected to ribosomal processes, such as rRNA processing and export, ribosomal subunit biogenesis and assembly, and amino acid synthesis (Fig. S2, Supporting Information). This was expected, as P*_TPI1_*-*STB5* had an approximately 40 and 20% lower maximum specific growth rate and biomass yield, respectively, compared to the control (Table S7, Supporting Information), and thus should require less protein synthesis. Simultaneously, gene sets connected to respiratory processes were stimulated by overexpression of *STB5* in the glucose phase (Fig. S2, Supporting Information), which correlated with the lower RQ value observed for P*_TPI1_*-*STB5* (Table S7, Supporting Information).

**Figure 6.**
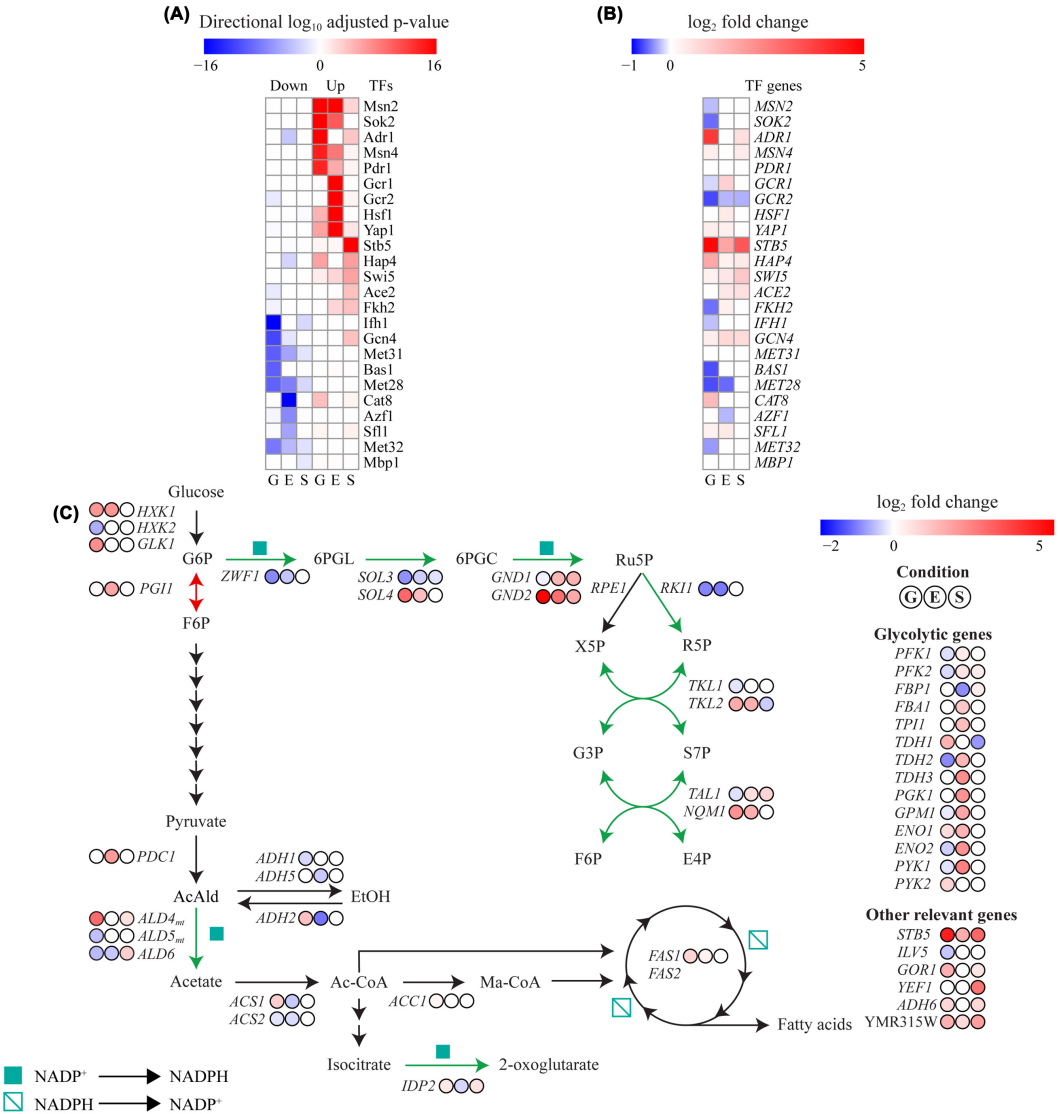
Transcriptional analysis of *STB5* overexpression in different conditions, where G, E and S correspond to the sampling point ‘Glucose’, ‘Ethanol’ and ‘Steady State’, respectively. A) TF reporter analysis, revealing the top five scored TFs predicted to drive the down- and upregulated transcriptional changes at each of the tested conditions. **B)** Differential expression of the reporter TF genes in respective conditions (padj < 0.01). **C)** Differential expression analysis of genes involved in the PPP, glycolysis, FA synthesis and other previously suggested *STB5* target genes (padj < 0.01).

We performed a reporter TF analysis to get a broader understanding if certain TFs were likely to be of importance for the overall transcriptional responses observed in the different growth stages. The result of the analysis highlights the top five reporter TFs related to a coordinated up- and downregulation of target genes in each condition, shown in Fig. 6A, and the LFC of each reported TF compared to the control in the same condition is presented in Fig. 6B. Adr1, essential for activation of glucose-repressed genes when cells enter the diauxic shift (Tachibana *et al*. 2005), stood out as a key TF in the glucose phase, together with Msn2, Msn4, Pdr1 and Sok2–transcriptional activators involved in stress response, pleiotropic drug resistance and cAMP-dependent protein kinase signal transduction. Of these, only *ADR1* showed a prominent degree of upregulation (Fig. 6B). In the study by Ouyang et al, *ADR1* and *MSN4* was indicated as direct targets of Stb5, although, *ADR1* was reported to be negatively regulated (Ouyang *et al*. 2018). Among other TFs which show a good correlation of target/TF expression is for example Ifh1, a ribosomal gene expression regulator, which is downregulated in the glucose phase as are its target genes. In the ethanol phase, *GCR1* encoding a transcriptional activator of genes involved in glycolysis is upregulated, which correlates well with a strong upregulation of majority of the glycolytic genes in the ethanol phase (Fig.   6C). Interestingly, Stb5 was only highlighted by the TF reporter analysis to have a significant positive influence on its target genes during steady state conditions, even though it was more strongly expressed in the glucose phase. This indicates that mechanisms are present to counteract the effects of Stb5-mediated regulation in glucose and ethanol phase. Furthermore, the other reporter TFs in steady state have lower significance compared to that observed for Stb5, indicating that fewer secondary effects were influencing expression compared to the situation in glucose and ethanol phase.

Figure 6C shows a selection of genes related to central carbon metabolism and previously indicated target genes of Stb5 and having significant altered expression (padj < 0.01). With respect to the genes of the PPP, which in a BY4741 background have been suggested to be activated by Stb5 in response to oxidative stress (Larochelle *et al*. 2006), all genes but two were differentially regulated in the glucose phase. *ZWF1*, *SOL3, RKI1, TAL1* and *TKL1* were significantly downregulated, the expression level of *GND1* and *RPE1* remained unchanged, while *SOL4*, *GND2, NQM1* and *TKL2* were induced. It should however be noted that *SOL4*, *GND2*, *NQM1* and *TKL2* had lower FPKM values than their corresponding paralogs in the glucose phase (Table S8, Supporting Information). When the raw read counts for each gene pair were added together and the combined LFC for *SOL*, *GND*, *TAL* and *TKL* were calculated with DESeq2, only *GND* has a slightly positive value while the other PPP reactions were affected negatively (significant LFC-values: -0.69, 0.18, -0.26 and -0.24, respectively), suggesting a reduced capacity for flux through the PPP in the glucose phase. In addition, the proposed effect of Stb5 being able to act as a transcriptional silencer of *PGI1* could not be seen in our study in any of the conditions.

Other NADPH-associated regulatory effects of Stb5 in response to oxidative stress in a BY4741 background included activation of *ALD6*, *IDP2*, *ILV5*, *GOR1*, *YEF1*, *ALD4* and *ADH6* (Larochelle *et al*. 2006). Among these, *ALD6* and *ILV5* were downregulated, *YEF1* remained unchanged while *IDP2*, *GOR1*, *ALD4* and *ADH6* were upregulated in response to *STB5* overexpression in the glucose phase. Even though *IDP2* had a positive LFC in the DE analysis, it is unlikely that it has a large influence on metabolism in the glucose phase due to its low FPKM value (Table S8, Supporting Information). *ALD4* was strongly upregulated, but the subcellular localization of the corresponding enzyme is within the mitochondria and the suggested redox shuttle gene *YHM2* was significantly downregulated (LFC = −1.31).

During growth on glucose, FA synthesis requires flux from pyruvate through the so-called pyruvate dehydrogenase (PDH) bypass, comprised by Pdc, Ald and Acs enzymes. *PDC*-gene expression was not significantly changed in the glucose phase. As mentioned in the section above, *ALD6*, responsible for the catalysis of acetaldehyde to acetate, was significantly downregulated in the glucose phase in strain P*_TPI1_*-*STB5*. The alcohol dehydrogenase Adh1 is the primary enzyme to divert flux from the PDH bypass, catalyzing the conversion of acetaldehyde to ethanol, while alcohol dehydrogenase Adh2 catalyzes the reverse reaction. Ethanol formation was reduced in strain P*_TPI1_*-*STB5* (Table S7, Supporting Information), also indicated by the reduced expression of *ADH1* (LCF = −0.39) and upregulation of *ADH2*. When the raw read counts for *ACS1* and *ACS2* were combined, a small negative effect on expression was observed (LFC = −0.22). With regards to the genes directely related to FA biosynthesis, *ACC1* and *FAS1* were significantly upregulated (LFC = 0.21 and LFC = 0.90).

In the ethanol phase, *STB5* overexpression led to an approximate 3-fold increase in *STB5* transcripts compared to the control, which is in agreement with a lower activity of the *TPI1* promoter under ethanol consumption (Peng *et al*. 2015). A large number of transcriptional processes were however affected, indicating that limited overexpression of *STB5* is enough to influence the expression of its target genes. As previously mentioned, *STB5* overexpression caused a coordinated upregulation of glycolytic genes (Fig. 6C) in the ethanol phase, which likely is mediated through an upregulation of the TF gene *GCR1* highlighted in the TF analysis (Fig. 6A and B), which is instrumental in the expression of glycolytic genes (Uemura and Fraenkel 2000). Simultaneously, gluconeogenic genes (*PDB1*, *PCK1* and *PYC1*) and the initial gene in the oxidative PPP (*ZWF1*) were downregulated compared to the control, indicating that flux through the oxidative PPP was reduced in the ethanol phase (Fig. 6C). *GND1*, *GND2*, *TAL1*, *NQM1* and *TKL2* were all significantly upregulated, suggesting that precursors for biosynthesis could be obtained from the non-oxidative PPP. Compared to growth on glucose, expression of NADPH-producing *IDP2* increased markedly in the ethanol phase for both strains (Table S8, Supporting Information) which correlates well with previous reports that its expression is induced in the presence of non-fermentable carbon sources (Haselbeck and Mcalisterhenn 1993). Nevertheless, both *IDP2* and *ALD6* were in the ethanol phase downregulated in the P*_TPI1_*-*STB5* strain compared to the control, while neither of *ILV5*, *GOR1*, *YEF1*, *ALD4* and *ADH6* were differentially expressed. Furthermore, a number of glyoxylate and TCA cycle genes were significantly downregulated (*ICL1*, *MLS1*, *MDH2*, *SFC1*, *LDC1*, *SDH3* and *FUM1*) in the ethanol phase. Gene-set analysis of differentially expressed genes in the ethanol phase (Fig. S3, Supporting Information) also highlights a distinct downregulation of processes such as cristae formation and ATP synthesis coupled proton transport, suggesting that the strain had a reduced respiratory capacity in the ethanol phase.

At steady state conditions, the overexpression mutant had a higher consumption of oxygen and production of carbon dioxide while the biomass yield was about 91% of that observed for the control (Table S7, Supporting Information), indicating a higher degree of respiration. These observations were also reflected in the transcriptional data, as gene set analysis shows that ribosomal processes were downregulated while respiratory and mitochondrial processes were upregulated (Fig. S4, Supporting Information). Within the PPP, transcription of *ZWF1*, *SOL4* and *RKI1* were not significantly changed, *SOL3* and *TKL2* were downregulated (LFC = −0.27 and -0.49, respectively), while *GND1*, *GND2*, *RPE1* and *TAL1* were upregulated (LFC = 1.64, 1.81, 0.22 and 0.89, respectively). Of the other genes indicated to be activated by Stb5 in the study of Larochelle *et al*. (2006), almost all were upregulated: *ALD6*, *IDP2*, *GOR1*, *YEF1*, *ALD4* and *ADH6* (LFC = 1.0, 0.61, 0.53, 2.96, 0.63 and 0.82). This agrees with the reporter TF analysis, which highlighted that Stb5 target genes were considered significantly upregulated in steady state conditions but not in glucose nor ethanol phase (Fig.   6A).

## DISCUSSION

The targeting of TFs has been attempted previously in metabolic engineering strategies in *S. cerevisiae*, mainly to abolish certain types of negative regulation, such as the deletion of *OPI1* to boost phospholipid synthesis (Feng, Lian and Zhao 2015). Fewer studies have investigated the overexpression of transcriptional activators to influence production, a likely result of the typically broad target range of TF.

We decided to evaluate overexpression of the TF *STB5* to influence FA biosynthesis, as previous reports had suggested it to have a primary role in the oxidative stress response via the transcriptional activation of genes related to NADPH generation (Larochelle *et al*. 2006; Hector *et al*. 2009; Ouyang *et al*. 2018). Previously, it has been shown that a rerouting of glycolytic flux through the oxidative PPP mediated by a coordinated upregulation of several PPP genes and downregulation of *PGI1* had a beneficial effect on FFA synthesis (Yu *et al*. 2018). Larochelle *et al*. reported that *STB5*, in part, is likely to mediate that particular effect when the cell is exposed to oxidative stress (Larochelle *et al*. 2006), which is why we were interested to know if a similar result could be obtained by simply replacing the native *STB5* promoter. If functional, this potentially could constitute an easy-to-implement strategy to boost NADPH supply for various applications.

Indeed, during the preparation of this manuscript, several recent reports were published exploring if overexpression of *STB5* could increase efficiency of biosynthetic processes dependent on NADPH or a high flux through the PPP (Hong *et al*. 2019; Kim *et al*. 2018; Park *et al*. 2018). Hong *et al*. observed that *STB5* and *OLE1* co-overexpression significantly increased lycopene titers in an engineered strain (Hong *et al*. 2019). Park *et al*. overexpressed *STB5* as a means to enhance the flux through the PPP, and found the strategy positively stimulated shinorine production (Park *et al*. 2018). However, even while Kim *et al*. observed a 1.2-fold increase in available NADPH upon *STB5* overexpression, the titer of their target-product protopanaxadiol was negatively affected (Kim *et al*. 2018). The results of these studies highlight the broad potential of *STB5* overexpression as a metabolic engineering strategy, but simultaneously raise questions about the systemic effects *STB5* overexpression may have on the host—a gap which our analysis seeks to fill.

In a previous study by Cadiere *et al*., *STB5* was overexpressed by replacement of the native promoter with P*_TDH3_*, which resulted in the strain not being able to grow on glucose, an effect that was attributed to the strain´s inability to re-oxidize a surplus of cellular NADPH (Cadiere, Galeote and Dequin 2010). In our study, we found that overexpression of *STB5* only resulted in a reduction in growth rate (Table 3) and final biomass concentration (Table S5, Supporting Information). This could be attributed to the use of different yeast strains, as Cadiere *et al*. used a wine yeast derivative likely to be dissimilar to CEN.PK113–5D. Even though we observed that a stronger expression was linked to a greater decrease in µ_max_, the difference was relatively small when comparing the effect of utilizing either P*_CYC1_* or P*_PGK1_*. P*_CYC1_* is considered to be a relatively weak promoter, and reduced µ_max_ to 80% of the value for the control, while the strong glycolytic promoter P*_PGK1_* resulted in a µ_max_ corresponding to 67% of the control. This could potentially be a result of target saturation—that even a limited overexpression of *STB5* successfully allows the TF to bind most of its target promoters and mediate transcriptional changes, while a stronger expression has a less pronounced effect. Our obtained RNAseq data show that the average FPKM of *STB5*, *CYC1* and *PGK1* in the control strain equals 16, 595 and 3015 in the glucose phase (Table S8, Supporting Information), respectively, which if directly translated to induction capacity of *STB5* supports that a saturation effect occurs. However, as the average FPKM of *TPI1* in the glucose phase was 2459 while it equaled 494 for *STB5* when it was placed under the control of P*_TPI1_*, a direct correlation between expression strength of the native genes and corresponding promoter-exchanged target cannot be made.

Our efforts to quantify cellular NADPH content in the exponential phase (Fig. S1, Supporting Information) did not deliver results resembling the outcome by Hector *et al*., who demonstrated a 2.5-fold increase in NADPH when *STB5* was overexpressed from a plasmid (Hector *et al*. 2009). Also in that particular study, a different yeast background was used (BY4727) compared to the one utilized herein (CEN.PK113–5D). Previous reports indicated that the NADP^+^/NADPH ratio is difficult to perturb in a CEN.PK background via genetic modifications. In response to overexpression of the NADPH-producing malic enzyme, CEN.PK113–7D counteracted imposed changes by reducing flux through the oxidative PPP, which resulted in an unchanged NADPH/NADP^+^ ratio (Moreira dos Santos *et al*. 2004). It is likely that *STB5*-overexpressing strains in our study counteracted a potentially increased NADPH synthesis with an increased consumption, for example by producing more FAs, (Fig. 3) or by partly overriding the *STB5*-mediated regulation by other mechanisms, such as transcriptionally downregulating key components of the PPP (Fig. 6C).

We also indirectly assessed NADPH supply in living cells by evaluating how well cells handle oxidative stress. For example, strains devoid of *ZWF1* and oxidative PPP flux have frequently been shown to be sensitive to oxidative agents, as was the case here (Fig. 2). Contradictory to our initial hypothesis, overexpression of *STB5* reduced resistance against oxidative stress in a similar way to that observed for the *zwf1*∆ mutant. Partly, this sensitivity could be related to a reduced oxidative PPP flux, indicated by the RNAseq data (Fig. 6C). In response to oxidative perturbations, the cell requires an immediate stabilization of its redox state, which has been shown to be dependent on metabolic reconfiguration—namely a dynamic redirection of glycolytic flux towards the oxidative PPP (Ralser *et al*. 2009). This rerouting is initiated via the inhibition of glyceraldehyde-3-phosphate dehydrogenase (GAPDH) in response to oxidative perturbation, which occurs within seconds of exposure and thus effectively blocks the glycolytic flux. In comparison, transcriptional adaptation of PPP-gene expression to oxidative stress occurs after about 4–7 min (Chechik *et al*. 2008), during which time a redox collapse would have detrimental effects on cell physiology and survival. Furthermore, the cellular oxidative stress response is dependent on parameters additional to NADPH supply, and overexpression of *STB5* for example significantly reduced expression of *GSH1*, encoding the initial step in glutathione biosynthesis, (LFC = −0.75), which possibly could limit survival in response to oxidative stress.

Even though the experiment of strain survival in response to oxidative stress indicated a potential reduction in NADPH supply, we observed positive effects of *STB5* overexpression on FA synthesis, specifically in the glucose phase (Fig. 3A). Based on the initial hypothesis that *STB5* overexpression could redirect flux through the oxidative PPP, it initially appeared logical as the largest flux diversion could be achieved when there is a steady inflow of glucose. However, when weighing in the reduced survival of P*_TPI1_*-*STB5* in response to oxidative stress (Fig. 2), that the beneficial effect on FFA synthesis was independent of flux through Zwf1 (Fig. 3B) and differential gene expression analysis results of PPP genes (Fig. 6C), the beneficial effect on FFA synthesis most likely has other causes, potentially mediated through an increased NADPH supply via other enzymatic routes. The RNAseq analysis showed that previously reported Stb5-target genes encoding NADPH-producing enzymes such as *ALD4*, *ADH6*, *IDP2*, *GOR1* and *YMR315W* were significantly upregulated while *ALD6* and *ILV5* were downregulated (Fig. 6C). An increased net activity of corresponding enzymes, and/or reduced consumption of NADPH through other reactions, would not have been of help during an oxidative perturbation, as it would cause an upstream blockage of the carbon flux at GAPDH, but it could still represent a source of NADPH for FA synthesis.

We made an interesting observation with regards to the origin of NADPH supply for FA synthesis in the control strain. There was a 50% reduction in FFA synthesis in the *zwf1*∆ strain at the end of the cultivation (Fig. 3B), which indicates that the oxidative PPP has a significant role in FA synthesis in *S. cerevisiae* during ethanol consumption. This observation is consistent with the fact that the majority of NADPH for FA synthesis in the oleaginous yeast *Yarrowia lipolytica* stems from the oxidative PPP (Wasylenko, Ahn and Stephanopoulos 2015). Interestingly, no negative effect of *zwf1*∆ on FFA production was observed during the exponential phase. This could potentially be attributed to a low requirement of NADPH and biosynthetic precursors during fermentative growth when the biomass yield is low, exemplified by the low Y_SX_ in exponential phase (0.157) compared to that observed in complete respiratory conditions during steady state (0.517) (Table S7, Supporting Information). Strong overexpression of *STB5* led to a negative effect on FA synthesis in the ethanol phase (Fig. 3A). Based on the observation made in the *zwf1*∆ strain, this could possibly be explained by the ethanol phase specific upregulation of genes related to glycolysis and reduced expression of gluconeogenic genes and *ZWF1* observed in the P*_TP11_*-*STB5* strain. Furthermore, a reduced expression of genes related to respiration was also observed, which should affect generation of ATP, also demanded in high quantities during FA synthesis.

Both mild (P*_CYC1_*) to strong (P*_ADH1_*/P*_ADH1_*/P*_PGK1_*) overexpression of *STB5* resulted in a relatively equal improvement on FA production (Fig. 3A), while the growth rate between the strains differed significantly (Table S6, Supporting Information). Thus, we wondered if NADPH supply was increased to a larger extent in strains where *STB5* expression was controlled by a strong promoter, but that the precursor acetyl-CoA instead could constitute a limitation for FA biosynthesis in the glucose phase. In order to study if this was the case, we employed a phosphoketolase pathway as shown by previous studies to enhance acetyl-CoA availability (Meadows *et al*. 2016). Initially, no beneficial effect on FA synthesis was observed when the Xfpk/Pta pathway was expressed in the control strain (Fig. 4, AB26 vs AB25). This is likely due to the efficient native conversion of acetyl phosphate to acetate via Gpp1 and Gpp2 (Bergman *et al*. 2016), also strengthening the hypothesis that the acetate to acetyl-CoA conversion is limited in glucose phase. Therefore, we decided to delete the gene encoding the phosphatase Gpp1 in the control and *STB5*-overexpression strain, and showed that this deletion alone had a positive effect on FA synthesis (AB27 vs AB25). Possibly, this could be related to the fact that glycerol-3-phosphate—which is likely to accumulate in the *gpp1*∆ strain—is a substrate for phospholipid and triacylglycerol (TAG) synthesis. Cellularly produced FFAs in yeast have been shown to stem from the hydrolysis of neutral lipids and phospholipids (Leber *et al*. 2014; Ferreira *et al*. 2018b). Expression of Xfpk/Pta in this background significantly increased FFA production by 25% (AB28 vs AB27), while its expression in the *gpp1*∆ P*_TPI1_*-*STB5* background increased FFA production by 37% (AB30 vs AB29). The increased acetyl-CoA supply provided by the Xfpk/Pta-pathway and consequences of *STB5* overexpression apparently had a synergistic effect on FFA synthesis, as the sum of the individual effects on FFA synthesis was lower than the observed dual effect. This would be an expected outcome if *STB5* overexpression were able to increase the NADPH supply, as a surplus of NADPH only will be efficiently utilized for FA synthesis if acetyl-CoA is readily available. However, the RNAseq analysis pointed out that expression of *ACC1* and *FAS1* was upregulated in response to *STB5* overexpression, which also could lead to a more efficient utilization of cytosolic acetyl-CoA. Unfortunately, the impact of these two separate effects cannot be resolved.

*STB5* overexpression by itself does not appear to be able to provoke the same gene expression changes in the glucose phase as observed when cells are treated with oxidative stress (Larochelle *et al*. 2006). Ouyang *et al*. investigated Stb5-mediated regulation in different environmental conditions in absence of oxidative stress agents, and showed that Stb5 was bound to the promoter of *ZWF1* in all instances, whereas deletion of *STB5* did not affect *ZWF1* transcription (Ouyang *et al*. 2018). However, when exposed to diamide, *ZWF1* induction has been shown to be strongly reduced in a *stb5*∆ strain compared to the control (Larochelle *et al*. 2006). Similarly, Hector *et al*. observed a moderate induction of a reporter gene placed under the control of a Stb5-regulated promoter by overexpressing *STB5*, while when treating the cells with the oxidative agent diamide, they observed a 2-fold increase in activity (Hector *et al*. 2009). Possibly, the cellular consequences brought about by oxidative stress, such as a reduced NADPH/NADP^+^  ratio, are required for proper binding of Stb5 to its binding motif or subsequent induction.

Additionally, NADPH produced by the cell will efficiently be consumed when exposed to oxidative stress—a redox sink which was not present in strain P*_TPI1_*-*STB5* (AB15). A perturbation of the redox balance would have global cellular effects and could potentially be a starting signal to the complex transcriptional reprogramming observed in P*_TPI1_*-*STB5* in the glucose phase. The maintenance of redox homeostasis typically involves to counteract a drop in the NADPH/NADP^+^ ratio, a situation which for example is commonly encountered during aerobic respiration when ROS form due to leakage of electrons from the respiratory chain (Aung-Htut *et al*. 2012). The reverse situation is likely to be a rare event in nature, but as mentioned previously, overexpression of a malic enzyme targeted to the cytosol was shown to decrease flux through the oxidative PPP (Moreira dos Santos *et al*. 2004). Furthermore, an increased cytosolic NADPH availability mediated via a deletion of a NADP^+^-dependent glutamate dehydrogenase gene *GDH1* was found to decrease transcription of *ZWF1*, *GND1* and *ALD6* (Bro, Regenberg and Nielsen 2004). This correlates well with the observed downregulation of *ZWF1* and *ALD6*, and the anticipated but not actualized Stb5-mediated upregulation of *GND1*, in strain P*_TPI1_*-*STB5* in exponential phase.

Furthermore, Ouyang *et al*. found, among others genes, *GND1*, *TAL1*, *ALD6* and *YEF1* to be bound by Stb5 in the control strain and simultaneously downregulated in a *stb5*∆ strain during glucose-limited conditions, indicating that Stb5 acts directly as a transcriptional activator of these genes in the absence of oxidative stress (Ouyang *et al*. 2018). Even though these genes were either not significantly changed or even downregulated during the exponential phase in our study, during chemostat conditions all of the mentioned genes were significantly upregulated in response to *STB5* overexpression (LFC corresponding to 1.64, 0.89, 1.02 and 2.96). Furthermore, repression of *ZWF1* transcription was not observed in steady state conditions, and all but one of the remaining target genes suggested by Larochelle *et al*. (*IDP2*, *GOR1*, *ALD4* and *ADH6*) were also upregulated. The cellular NADPH/NADP^+^ ratio is generally held high in order to be able to drive biosynthetic reactions, and the cytosolic ratio in CEN.PK 113–7D has been reported to be 22.0 and 15.6 during exponential phase and steady state, respectively (Zhang *et al*. 2015). This supports that an increase in NADPH supply would be particularly damaging in the exponential phase, which could be an explanation for the larger reduction in biomass yield observed for strain P*_TPI1_*-*STB5* in the exponential phase (81% of control) compared to steady state (91% of control) (Table S7, Supporting Information). Taken together, this strongly implies that the Stb5-mediated gene regulation partially is superseded by stronger regulatory mechanisms to counteract a redox imbalance in the batch phase.

We observed a mitigation of the negative influence of mild *STB5* overexpression (promoter P*_CYC1_*) had on growth rate when a strain with a greater ability for FA synthesis was utilized (Table 3 vs Table S6, Supporting Information). With this in mind, optimally a mild to moderate overexpression of *STB5* in a yeast strain with excellent ability to consume the NADPH during product formation is suggested for optimal performance. This would likely increase biosynthetic ability of the desired compound while at the same time reducing undesirable systemic effects. Furthermore, as negative effects on FFA synthesis were observed in the ethanol phase, the utilization of a glucose-specific promoter could be of interest to evaluate.

## Supplementary Material

foz027_Supplement_File
