# Supplementary material for: Effects of overexpression of STB5 in Saccharomyces cerevisiae on fatty acid biosynthesis, physiology and transcriptome
Source: FEMS Yeast Res. 2019 Mar 29;19(3):foz027. doi: 10.1093/femsyr/foz027 (PMC6755256; doi:10.1093/femsyr/foz027)
Supplement: foz027_Supplement_File [file foz027_Supplement_File.docx]

**Supplementary information**

**Supplementary figures**

**Figure S1.** Quantification of NADPH in control and promoter exchanged strains in CEN.PK 113-5D background. Strains were grown in 2% glucose and harvested in early exponential phase. Values are the average of biological triplicates, error bars ± standard deviation.


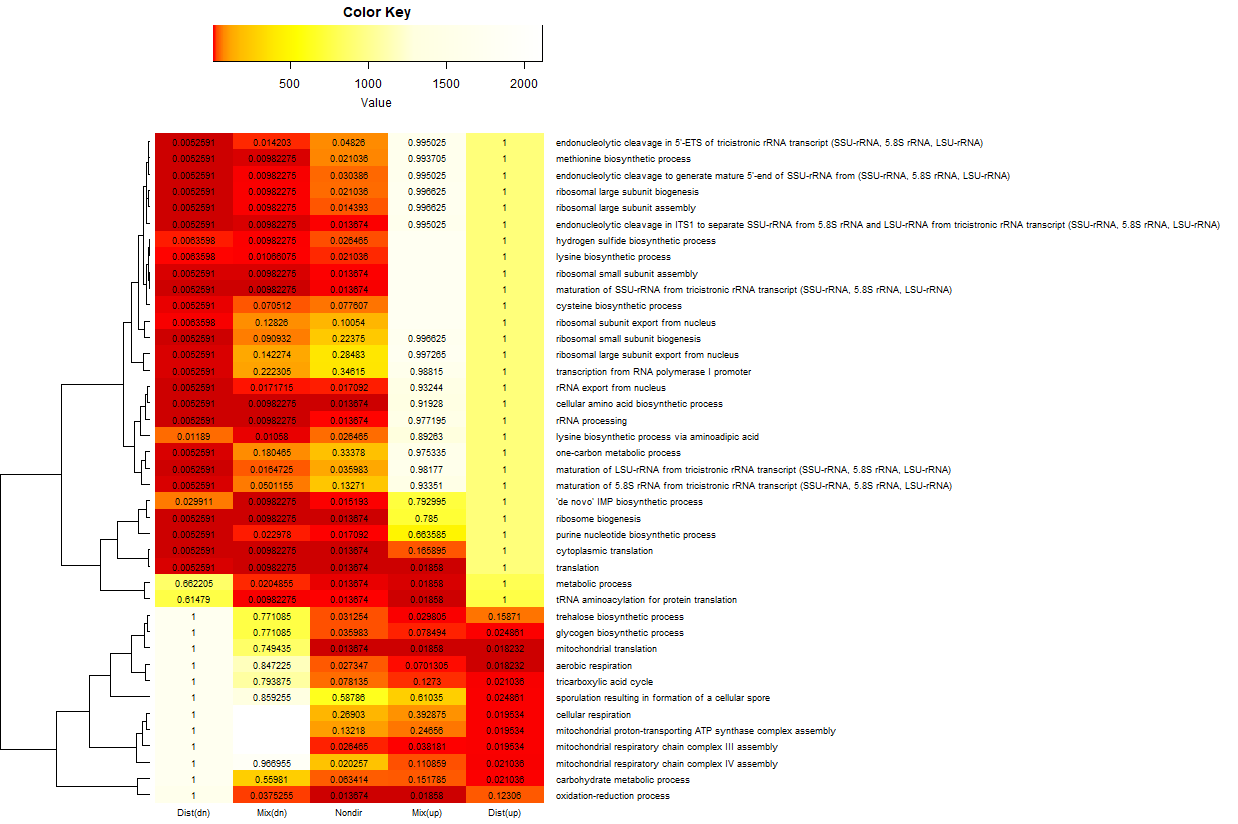


**Figure S2.** Consensus heat map of gene set analysis of *STB5* overexpression (P*_TPI_*-*STB5*) compared to the control in glucose phase. The number in each cell is the median adjusted p-value from different GSA methods (mean, median, sum, mazmean, fisher, stouffer, tailStrength, gsea and page). The color key is the consensus rank (mean rank of each gene set from each GSA method). See “Varemo L, Nielsen J, Nookaew I. Enriching the gene set analysis of genome-wide data by incorporating directionality of gene expression and combining statistical hypotheses and methods. Nucleic Acids Res. 2013;41(8):4378-91” for details.

**
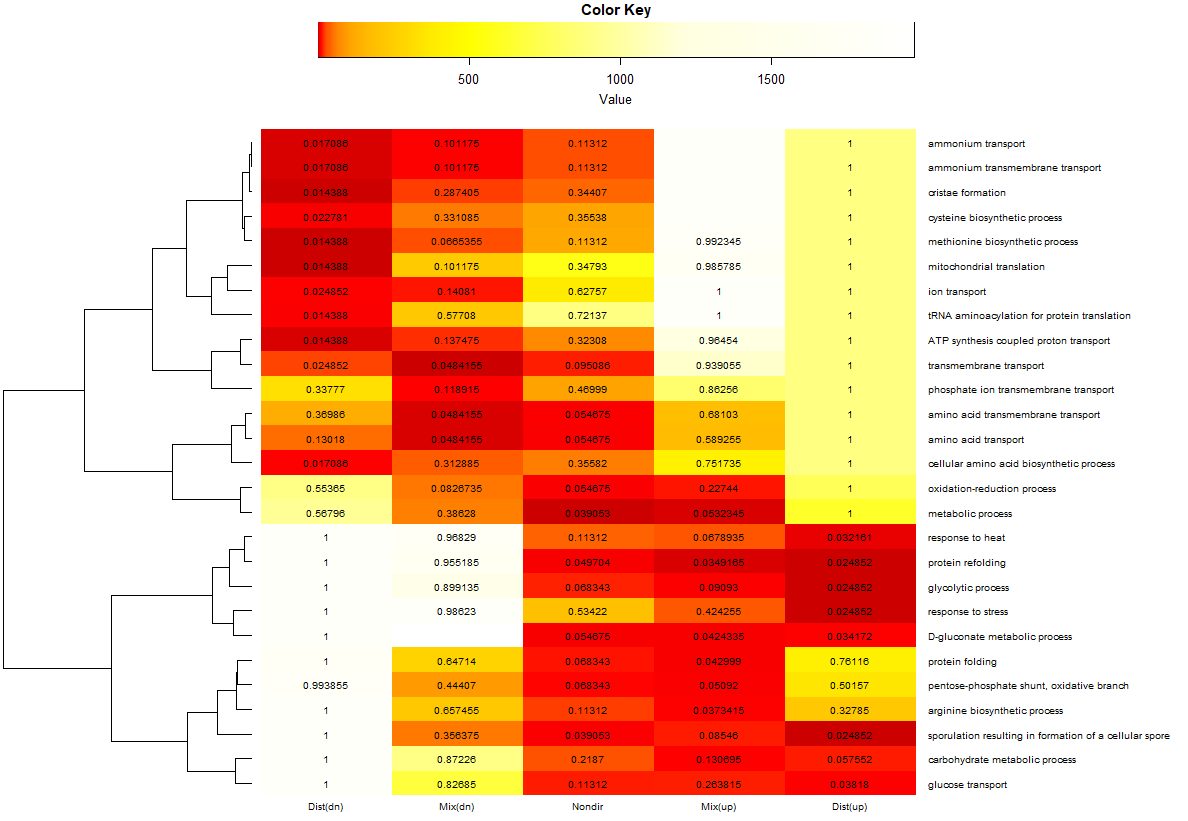
**

**Figure S3.** Consensus heat map of gene set analysis of *STB5* overexpression (P*_TPI_*-*STB5*) compared to the control in ethanol phase. The number in each cell is the median adjusted p-value from different GSA methods (mean, median, sum, mazmean, fisher, stouffer, tailStrength, gsea and page). The color key is the consensus rank (mean rank of each gene set from each GSA method). See reference stated in legend of Figure S2 for details.

**
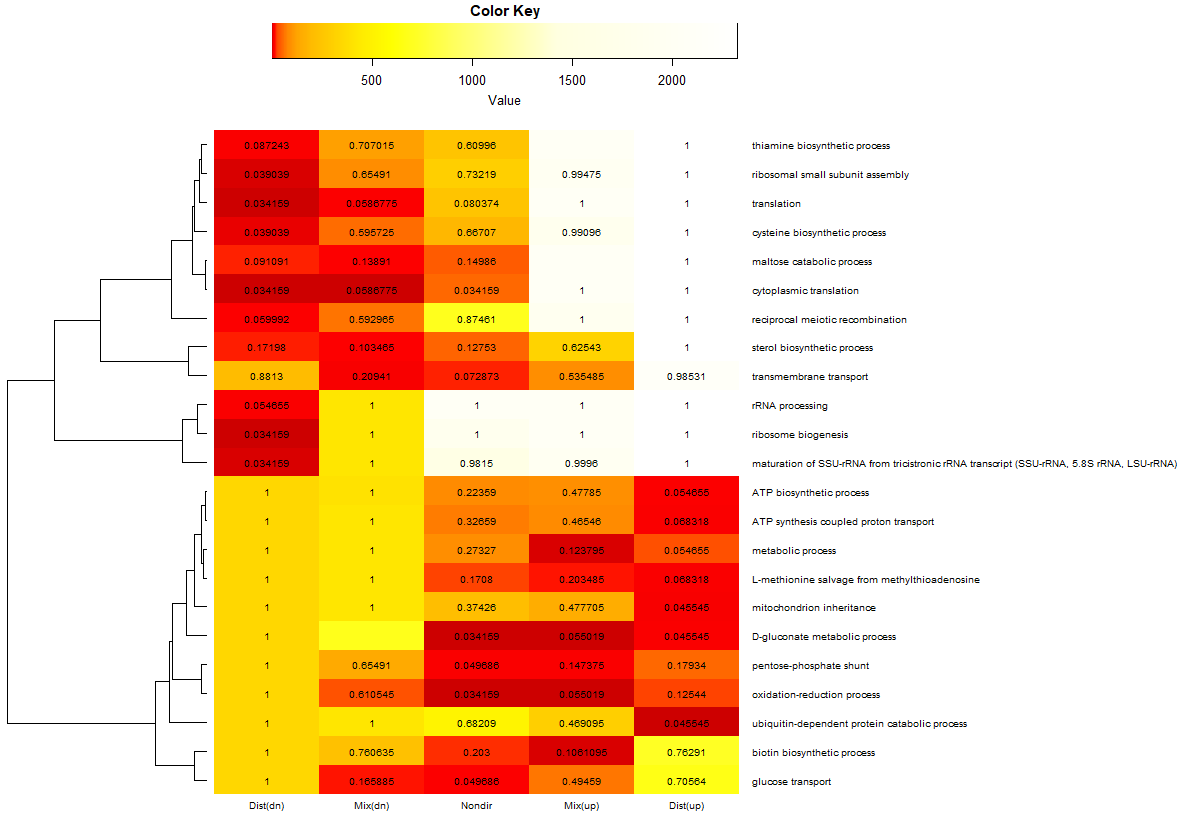
**

**Figure S4.** Consensus heat map of gene set analysis of *STB5* overexpression (P*_TPI_*-*STB5*) compared to the control in steady state conditions. The number in each cell is the median adjusted p-value from different GSA methods (mean, median, sum, mazmean, fisher, stouffer, tailStrength, gsea and page). The color key is the consensus rank (mean rank of each gene set from each GSA method). See reference stated in legend of Figure S2 for details.

**Supplementary tables**

**Table S1.** Guide-RNA sequences.

| **Target** | **gRNA sequence** |
| --- | --- |
| *FAA1* | TTCTTAGGAGCGCAGTCTCA |
| *FAA4* | ATGTTCAGGCTCCAGGACAC |
| *ZWF1* | GCAGTACAAAGCGGTCGCAT |
| *GPP1* | CACCTCTGGTACCCGTGACA |
| *STB5* | CCGCAGGGAATATAACGACA |

**Table S2.** Primers used in this study and description of amplification strategy.

**A)** For the first PCR reaction, the primers 1/5/7/9 were used with 12 and primers 4/6/8/10 were used with 11, while primer 3 was used with 14 and primer 2 with 13. The resulting fragments corresponding to genes *ZWF1*, *GPP1*, *STB5* were fused using primers 11 and 12, while *FAA1* and *FAA4* fragments were fused with primer 11 and 14 and 12 and 13, respectively. Primer 15 and 16 were used to verify gRNA insertion(s) and to amplify inserted cassette(s) for DNA sequencing.

**B)** The up- and downstream DNA sequences targeting the *STB5* promoter were amplified with primers 17/18 and 19/20, and the promoters of *CUP1*, *CYC1*, *ADH1*, *ADH1*, *TPI1* and *PGK1* were amplified with primer pairs 21/22, 23/24, 25/26, 27/28 and 29/30, respectively. The up- and downstream *STB5* fragments were fused with the amplified promoters using primers 17/20. Primers 31/32 were used to amplify the region of the promoter replacement, which were confirmed by sequencing.

**C)** Oligonucleotide pairs 33/34, 35/36, 37/38 and 39/40 were annealed by heating a 50:50 mixture of 100 µM oligo solutions to 99°C for 5 minutes and letting them re-anneal at room temperature, and used as repair fragment in CRISPR transformation for deletions. Deletions were verified using primer pairs 41/42, 43/44, 45/46 and 47/48.

**D)** The *PGK1* promoter and *CYC1* terminator of pSP-GM1 and the codon optimized phosphotransacetylase gene from *Clostridium kluyveri* were amplified with primer pairs 49/50, 51/52 and 53/54, respectively. The three fragments were fused using primers 49/54.

| **A. Primers for gRNA cassette amplification and plasmid verification** | | |
| --- | --- | --- |
| **No.** | **Primer** | **Sequence** |
| 1 | F-gRNA.FAA1 | TTCTTAGGAGCGCAGTCTCAGTTTTAGAGCTAGAAATAGCAAG |
| 2 | R-gRNA.FAA1 | TGAGACTGCGCTCCTAAGAAGATCATTTATCTTTCACTGCGGA |
| 3 | F-gRNA.FAA4 | ATGTTCAGGCTCCAGGACACGTTTTAGAGCTAGAAATAGCAAG |
| 4 | R-gRNA.FAA4 | GTGTCCTGGAGCCTGAACATGATCATTTATCTTTCACTGCGGA |
| 5 | F-gRNA.ZWF1 | GCAGTACAAAGCGGTCGCATGTTTTAGAGCTAGAAATAGCAAG |
| 6 | R-gRNA.ZWF1 | ATGCGACCGCTTTGTACTGCGATCATTTATCTTTCACTGCGGA |
| 7 | F-gRNA.GPP1 | CACCTCTGGTACCCGTGACAGTTTTAGAGCTAGAAATAGCAAG |
| 8 | R-gRNA.GPP1 | TGTCACGGGTACCAGAGGTGGATCATTTATCTTTCACTGCGGA |
| 9 | F-gRNA.STB5 | CCGCAGGGAATATAACGACAGTTTTAGAGCTAGAAATAGCAAG |
| 10 | R-gRNA.STB5 | TGTCGTTATATTCCCTGCGGGATCATTTATCTTTCACTGCGGA |
| 11 | F-HR.pCas9 | TAGGCGTATCACGAGATCGCGTCAGCTGAAGCTTC-TCTTTGAAAAGATAATGTATGATTATGC |
| 12 | R-HR.pCas9 | ATACGAAGTTATATTAAGGGTTGTCGACCTGCAGC-AGACATAAAAAACAAAAAAAGCACC |
| 13 | F-gRNA.fuse | GTCGGATCTGAGTCGACCAATCTTTGAAAAGATAATGTATGATTATGC |
| 14 | R-gRNA.fuse | TTGGTCGACTCAGATCCGACAGACATAAAAAACAAAAAAAGCACC |
| 15 | F-Ver.pCas9 | CAAGACTGTCAAGGAGGGTA |
| 16 | R-Ver.pCas9 | AGATACGAGGCGCGTGTAAG |
| **B. Primers for promoter insertions** | | |
| **No.** | **Primer** | **Sequence** |
| 17 | F-STB5.UP | CACCTTTCTCACGTAGTTAAAGTTGCAAC |
| 18 | R-STB5.UP | GGATCTGATATACCGTTCGTATAGCTCATGCCTTCTTGTCTTAGAACAT |
| 19 | F-STB5.DW | ATGGATGGTCCCAATTTTGCACATC |
| 20 | R-STB5.DW | TGCGGATAGCGGACTAGAAGAGTTT |
| 21 | F-CUP1 | GCTATACGAACGGTATATCAGATCC-  TTTAAAACACTTTTGTATTATTTTTCCTCA |
| 22 | R-CUP1 | GATGTGCAAAATTGGGACCATCCATTGTTTTTTTATGTGATGATTGATTG |
| 23 | F-CYC1 | GCTATACGAACGGTATATCAGATCCTGAGACGACATCGTCGAATATGATT |
| 24 | R-CYC1 | GATGTGCAAAATTGGGACCATCCATTATTAATTTAGTGTGTGTATTTGTG |
| 25 | F-ADH1 | GCTATACGAACGGTATATCAGATCCgatatccttttgttgtttccgggtg |
| 26 | R-ADH1 | GATGTGCAAAATTGGGACCATCCATGTATATGAGATAGTTGATTGTATG |
| 27 | F-TPI1 | GCTATACGAACGGTATATCAGATCC-  CTACGTATGGTCATTTCTTCTTCAG |
| 28 | R-TPI1 | GATGTGCAAAATTGGGACCATCCAT-  TTTTAGTTTATGTATGTGTTTTTTGTAGTT |
| 29 | F-PGK1 | GCTATACGAACGGTATATCAGATCC-  AGAAGTACCTTCAAAGAATGGGGTC |
| 30 | R-PGK1 | GATGTGCAAAATTGGGACCATCCAT-  TTGTTTTATATTTGTTGTAAAAAGTAGATA |
| 31 | F-STB5.Ver | GAACCCCAATCAAAACCAT |
| 32 | R-STB5.Ver | CATCCGCTAACTCCTTCT |
| **C. Oligonucleotides for gene deletions** | | |
| **No.** | **Primer** | **Sequence** |
| **33** | **F-faa1∆** | AAACTCGTTAGGATACAATAAAAACTAGAACAAACACAAAAGAC-AAAAAAAGACAACAATTGGATCAACATTTCCATGATAGGAAAGC-  CTCATCATACTAAAGCACTTTTTCAGTTTTTT |
| **34** | **R-faa1∆** | AAAAAACTGAAAAAGTGCTTTAGTATGATGAGGCTTTCCTATCAT-  GGAAATGTTGATCCAATTGTTGTCTTTTTTTGTCTTTTGTGTTTGTT-  CTAGTTTTTATTGTATCCTAACGAGTTT |
| **35** | **F-faa4∆** | CACATCATTTTTTTCTCTGTTCTTCACTATTTCTTGAAAAACTAAG-  AAGTACGCATCAAAAGGAAGACATAGTTTTTTACTTTCCCCCCTG-  CCCTTCATAAACACTACGTTTCATTTTCT |
| **36** | **R-faa4∆** | AGAAAATGAAACGTAGTGTTTATGAAGGGCAGGGGGGAAAGT-  AAAAAACTATGTCTTCCTTTTGATGCGTACTTCTTAGTTTTTCAA-  GAAATAGTGAAGAACAGAGAAAAAAATGATGTG |
| **37** | **F-zwf1∆** | CAATTGGCTGTATAGACAGAAAGAGTAAATCCAATAGAATAGAA-  AACCACATAAGGCAAGAAAAATGCAAGCACATTCATTTATCGGCT-  AAGTCACTGAAATTTTTTTTTTCGAGTGATT |
| **38** | **R-zwf1∆** | AATCACTCGAAAAAAAAAATTTCAGTGACTTAGCCGATAAATGAA-  TGTGCTTGCATTTTTCTTGCCTTATGTGGTTTTCTATTCTATTGGATT-  TACTCTTTCTGTCTATACAGCCAATTG |
| **39** | **F-gpp1∆** | AAACGTTTCAATGTTTTAAAATATATCAGAACAACAAAAGCAAATA-  TACAAACCATCGCACTAAGGATGACTTGTTGAAATGGTAATTTTCTT-  TTATTTTTTTGATAAAACTACTACGCT |
| **40** | **R-gpp1∆** | AGCGTAGTAGTTTTATCAAAAAAATAAAAGAAAATTACCATTTCAAC-  AAGTCATCCTTAGTGCGATGGTTTGTATATTTGCTTTTGTTGTTCTGAT-  ATATTTTAAAACATTGAAACGTTT |
| **41** | **F-faa1∆.Ver** | TACGAATGACACAGGGGCAC |
| **42** | **R-faa1∆.Ver** | AAGGAGTCAGTGCACACCAG |
| **43** | **F-faa4∆.Ver** | TGCAACCCCTCTGAGTTGAC |
| **44** | **R-faa4∆.Ver** | TGCATAAATGAACGTGGGCG |
| **45** | **F-zwf1∆.Ver** | CAATAATAGTAGCGCTACTGG |
| **46** | **R-zwf1∆.Ver** | CTCCTCTATAATGGCATCTTC |
| **47** | **F-gpp1∆.Ver** | CCTTTGATGATGTGTGAGTT |
| **48** | **R-gpp1∆.Ver** | GAAGTCTGAAAAGAAGTCTA |
| **D. Primers for pAB-XP plasmid construction** | | |
| **No.** | **Primer** | **Sequence** |
| **49** | **F-PGK1(P)** | CAATTTCGTCACACAACAAG |
| **50** | **R-PGK1(P)** | TTGTTTTATATTTGTTGTAAAAAGTAGATA |
| **51** | **F-CYC1(T)** | ATCCGCTCTAACCGAAAAG |
| **52** | **R-CYC1(T)** | CATGTTCTTTCCTGCGTTAT |
| **53** | **F-PTA(CK)** | TATCTACTTTTTACAACAAATATAAAACAAAAAACA-ATGAAGTTGATGGAAAACATCTTCG |
| **54** | **R-PTA(CK)** | CTAACTCCTTCCTTTTCGGTTAGAGCGGAT-TTAACCTTGTGCTTGAGCTTGAACG |

**Table S3.** Gene sequence of phosphotransacetylase from *Clostridium kluyveri*, codon optimized for *S. cerevisiae*

| atgaagttgatggaaaacatcttcggtttggccaaggctgataaaaagaaaatagttttggctgaaggtgaagaagaaagaaacatcagagcatccgaagaaattataagagacggtattgccgatatcattttggttggttccgaaagtgtcatcaaggaaaatgctgcaaaattcggtgttaacttagctggtgttgaaatagtcgatccagaaacttcttcaaaaacagcaggttacgcaaacgccttctacgaaatcagaaagaacaaaggtgttacattagaaaaggccgacaaaatagtcagagatcctatctatttcgcaaccatgatggttaagttgggtgacgccgacggtttagtttcaggtgctattcatactactggtgacttgttaagaccaggtttgcaaatagtaaaaactgttcctggtgcttccgttgtctccagtgtatttttaatgagtgttccagactgtgaatacggtgaagatggtttcttgttattcgccgattgtgctgtcaatgtatgccctaccgcagaagaattgtcttcaatagccatcaccactgctgaaactgccaagaacttgtgcaaaattgaaccaagagttgctatgttatctttctcaacaatgggttccgcaagtcacgaattagtcgataaagtaacaaaagctaccaagttggctaaagaagcaagaccagatttggatattgatggtgaattgcaattagacgcatctttggttaagaaagtcgctgatttgaaagcaccaggttcaaaggtcgccggtaaagctaatgtattgatctttcctgacattcaagctggtaacatcggttacaagttagttcaaagattcgctaaagcagaagccattggtccaatatgtcaaggtttcgctaagcctataaatgatttgtcaagaggttgttctgtcgatgacatcgtaaaagtagttgcagtaactgccgttcaagctcaagcacaaggttaa |
| --- |

**Table S4.** Version number and references for programs used in the NGI-RNAseq pipeline (<https://github.com/SciLifeLab/NGI-RNAseq>).

| **Program** | **Version** | **Reference** |
| --- | --- | --- |
| FastQC | 0.11.7 |  |
| Trim Galore! | 0.4.4 |  |
| Star | 2.5.3a | Dobin A, Davis CA, Schlesinger F, Drenkow J, Zaleski C, Jha S, et al. STAR: ultrafast universal RNA-seq aligner. Bioinformatics. 2013;29(1):15–21 |
| featureCounts | 1.6.0 | Liao Y, Smyth GK, Shi W. featureCounts: an efficient general purpose program for assigning sequence reads to genomic features. Bioinformatics. 2014;30(7):923–30 |
| StringTie | 1.3.3 | Pertea M, Pertea GM, Antonescu CM, Chang T-C, Mendell JT, Salzberg SL. StringTie enables improved reconstruction of a transcriptome from RNA-seq reads. Nat Biotechnol. 2015;33(3):290–5 |
| RSeQC | 2.6.4 | Wang L, Wang S, Li W. RSeQC: quality control of RNA-seq experiments. Bioinformatics. 2012;28(16):2184–5 |
| dupradar | 1.8.0 | Sayols S, Scherzinger D, Klein H. dupRadar: a Bioconductor package for the assessment of PCR artifacts in RNA-Seq data. BMC Bioinformatics. 2016;17(1):428. |
| Preseq | 2.0.1 | Daley T, Smith AD. Predicting the molecular complexity of sequencing libraries. Nat Methods. 2013;10(4):325–7. |
| Picard MarkDuplicates | 2.18.0 |  |
| edgeR | 3.20.1 | Robinson MD, McCarthy DJ, Smyth GK. edgeR: a Bioconductor package for differential expression analysis of digital gene expression data. Bioinformatics. 2010;26(1):139–40 |
| MultiQC | 1.5 | Ewels P, Magnusson M, Lundin S, Käller M. MultiQC: summarize analysis results for multiple tools and samples in a single report. Bioinformatics. 2016;32(19):3047–8 |
| nextflow | 0.29.0 | Di Tommaso P, Chatzou M, Floden EW, Barja PP, Palumbo E, Notredame C. Nextflow enables reproducible computational workflows. Nat Biotechnol. 2017;35(4):316–9 |

**Table S5.** Final cell density (OD_600_) of *STB5*-promoter exchanged strains in CEN.PK113-5D background.

| ***A)* STB5 *overexpression mediated by copper induction*** | | | | |
| --- | --- | --- | --- | --- |
| **Cu^2+^ (µM)** | **Control (CEN.PK 113-5D)** | | **P*_CUP1_*-*STB5* (AB12)** | |
| **"0"** | 11.55 ± 0.30 | | 12.07 ± 0.26 | |
| **100** | 12.20 ± 0.23 | | 10.64 ± 0.11 | |
| **200** | 11.94 ± 0.22 | | 10.41 ± 0.19 | |
| **400** | 12.05 ± 0.23 | | 9.62 ± 0.21 | |
| ***B)* STB5 *overexpression mediated by constitutive promoters*** | | | | |
| **Control**  **(CEN.PK 113-5D)** | **P*_CYC1_*-*STB5***  **(AB13)** | **P*_ADH1_*-*STB5***  **(AB14)** | **P*_TPI1_*-*STB5***  **(AB15)** | **P*_PGK1_*-*STB5***  **(AB16)** |
| 12.03±0.28 | 10.50±0.19 | 9.51±0.24 | 9.38±0.17 | 9.43±0.16 |

Strains were grown in minimal media with 2% glucose and 60 mg/L uracil. “0 µM” Cu^2+^ concentration in reality corresponds to approximately 6 µM due to the presence of Cu^2+^ in the trace metal solution added to the media. Values correspond to the average of biological triplicates ± standard deviation.

**Table S6.** Growth rate of *STB5*-promoter exchanged strains in CEN.PK 113-5D *faa1∆faa4∆*. Significant changes (p<0.05) are indicated with an asterisk.

| **Strain** | **µ_max_ (h^-1^)** |
| --- | --- |
| Control (AB11) | 0.397 ± 0.005 |
| P*_CYC1_*-*STB5* (AB17) | 0.386 ± 0.017 |
| P*_ADH1_*-*STB5* (AB18) | 0.260 ± 0.007 * |
| P*_TPI1_*-*STB5* (AB19) | 0.285 ± 0.010 * |
| P*_PGK1_*-*STB5* (AB20) | 0.256 ± 0.008 * |

Strains were cultivated in 2% glucose. Values are average of biological triplicates ± standard deviation.

**Table S7.** Physiological parameters of control and P*_TPI1_*-*STB5* strains in CEN.PK 113-5D background in exponential phase and steady state when cultivated in bioreactors.

|  | **Exponential phase** | | **Steady state** | |
| --- | --- | --- | --- | --- |
|  | Control | P*_TPI1_*-*STB5* | Control | P*_TPI1_*-*STB5* |
| µ_max_ (h^-1^) | 0.392 ± 0.017 | 0.251 ± 0.01 | 0.092 ± 0.003 | 0.092 ± 0.004 |
| Y_S/X_ (g g^-1^) | 0.157 ± 0.012 | 0.128 ± 0.006 | 0.517 ± 0.014 | 0.472 ± 0.011 |
| q_Glucose_ (mmol gCDW^-1^ h^-1^) | -13.69 ± 0.71 | -10.52 ± 0.86 | -1 ± 0.06 | -1.09 ± 0.05 |
| q_Glycerol_ (mmol gCDW^-1^ h^-1^) | 1.46 ± 0.1 | 0.73 ± 0.1 | - | - |
| q_Acetate_ (mmol gCDW^-1^ h^-1^) | 0.29 ± 0.03 | 0.27 ± 0.03 | - | - |
| q_Ethanol_ (mmol gCDW^-1^ h^-1^) | 15.73 ± 1.32 | 9.93 ± 1.23 | - | - |
| q_Biomass_ (mmol gCDW^-1^ h^-1^) | 15.91 ± 0.7 | 10.2 ± 0.44 | 3.72 ± 0.14 | 3.72 ± 0.16 |
| q_CO2_ (mmol gCDW^-1^ h^-1^) | 34.32 ± 1.69 | 23.45 ± 1.48 | 2.73 ± 0.26 | 2.89 ± 0.1 |
| q_O2_ (mmol gCDW^-1^ h^-1^) | 2.26 ± 0.6 | 2.82 ± 0.44 | 2.18 ± 0.23 | 2.42 ± 0.12 |
| Carbon balance | 106% ± 7% | 89% ± 6% | 108% ± 2% | 101% ± 1% |
| RQ | 16.51 ± 5.09 | 8.48 ± 1.15 | 1.28 ± 0.25 | 1.2 ± 0.09 |

Glucose concentration was 2% during exponential phase, and 0.75% in the feed during chemostat mode. Values presented are average of biological quadruplicates ± standard deviation.

**Table S8.** FPKM values of selected genes for control and P*_TPI1_*-*STB5* strain (STB5) in glucose (G), ethanol (E) and steady state (S). The values are the mean of four technical replicates ± standard deviation.

|  | **Control(G)** | **STB5(G)** | **Control(E)** | **STB5(E)** | **Control(S)** | **STB5(S)** |
| --- | --- | --- | --- | --- | --- | --- |
| *STB5* | 16 ± 1 | 494 ± 8 | 27 ± 2 | 87 ± 4 | 26 ± 2 | 235 ± 19 |
| **Pentose phosphate pathway** | | | | | | |
| *ZWF1* | 431 ± 26 | 210 ± 11 | 375 ± 31 | 234 ± 17 | 373 ± 12 | 351 ± 16 |
| *SOL3* | 308 ± 23 | 158 ± 9 | 52 ± 5 | 36 ± 6 | 242 ± 26 | 184 ± 11 |
| *SOL4* | 5 ± 1 | 49 ± 5 | 222 ± 31 | 551 ± 108 | 221 ± 24 | 185 ± 22 |
| *GND1* | 1033 ± 35 | 1019 ± 20 | 73 ± 4 | 204 ± 33 | 556 ± 72 | 1583 ± 216 |
| *GND2* | 5 ± 1 | 247 ± 9 | 97 ± 16 | 777 ± 140 | 445 ± 33 | 1437 ± 149 |
| *RPE1* | 242 ± 12 | 247 ± 18 | 256 ± 13 | 235 ± 13 | 207 ± 10 | 222 ± 17 |
| *RPI1* | 67 ± 6 | 70 ± 6 | 31 ± 5 | 61 ± 5 | 55 ± 7 | 45 ± 4 |
| *TKL1* | 603 ± 17 | 545 ± 23 | 161 ± 15 | 132 ± 59 | 266 ± 62 | 307 ± 20 |
| *TKL2* | 1 ± 1 | 5 ± 1 | 128 ± 27 | 399 ± 78 | 283 ± 36 | 183 ± 30 |
| *TAL1* | 949 ± 49 | 821 ± 20 | 333 ± 13 | 475 ± 32 | 961 ± 20 | 1626 ± 188 |
| *NQM1* | 5 ± 1 | 30 ± 9 | 226 ± 27 | 692 ± 114 | 447 ± 64 | 491 ± 44 |
| **Glycolysis** | | | | | | |
| *PFK1* | 402 ± 16 | 346 ± 14 | 109 ± 4 | 135 ± 11 | 214 ± 9 | 219 ± 10 |
| *PFK2* | 317 ± 16 | 264 ± 11 | 71 ± 2 | 100 ± 5 | 194 ± 6 | 233 ± 19 |
| *FBP1* | 1 ± 1 | 3 ± 1 | 2918 ± 175 | 1279 ± 99 | 116 ± 14 | 141 ± 7 |
| *FBA1* | 9040 ± 421 | 9181 ± 474 | 738 ± 97 | 1592 ± 103 | 5555 ± 689 | 6616 ± 661 |
| *TPI1* | 2441 ± 176 | 2459 ± 186 | 189 ± 13 | 466 ± 47 | 1053 ± 154 | 1229 ± 46 |
| *TDH1* | 113 ± 9 | 311 ± 41 | 255 ± 43 | 300 ± 60 | 582 ± 70 | 268 ± 85 |
| *TDH2* | 4620 ± 205 | 2294 ± 112 | 60 ± 4 | 170 ± 11 | 663 ± 44 | 525 ± 51 |
| *TDH3* | 8765 ± 394 | 8755 ± 486 | 677 ± 63 | 3330 ± 602 | 6188 ± 483 | 7407 ± 655 |
| *PGK1* | 3016 ± 118 | 3214 ± 225 | 177 ± 11 | 817 ± 126 | 2038 ± 229 | 1957 ± 165 |
| *GPM1* | 6191 ± 365 | 5843 ± 299 | 199 ± 8 | 716 ± 62 | 3048 ± 174 | 3062 ± 360 |
| *ENO1* | 487 ± 22 | 853 ± 72 | 80 ± 8 | 230 ± 37 | 1174 ± 127 | 989 ± 89 |
| *ENO2* | 4889 ± 288 | 3811 ± 282 | 30 ± 5 | 146 ± 19 | 1367 ± 60 | 1421 ± 131 |
| *PYK1* | 5129 ± 280 | 4598 ± 363 | 62 ± 6 | 377 ± 27 | 2192 ± 141 | 2114 ± 202 |
| *PYK2* | 17 ± 3 | 33 ± 2 | 54 ± 4 | 54 ± 6 | 26 ± 2 | 27 ± 3 |
| **PDH bypass/Fatty acid synthesis** | | | | | | |
| *PDC1* | 5767 ± 256 | 5625 ± 371 | 56 ± 5 | 239 ± 70 | 1814 ± 246 | 1452 ± 315 |
| *PDC5* | 13 ± 2 | 13 ± 1 | 12 ± 2 | 11 ± 2 | 11 ± 2 | 9 ± 2 |
| *PDC6* | 3 ± 1 | 5 ± 1 | 26 ± 4 | 17 ± 2 | 72 ± 34 | 12 ± 2 |
| *ADH1* | 4962 ± 306 | 4064 ± 215 | 357 ± 18 | 345 ± 47 | 1143 ± 33 | 1023 ± 96 |
| *ADH2* | 57 ± 3 | 48 ± 4 | 1977 ± 249 | 735 ± 82 | 5110 ± 1577 | 7930 ± 998 |
| *ADH5* | 92 ± 8 | 105 ± 13 | 111 ± 12 | 72 ± 6 | 43 ± 5 | 37 ± 4 |
| *ALD4* | 44 ± 4 | 427 ± 29 | 725 ± 72 | 712 ± 25 | 6172 ± 949 | 8797 ± 946 |
| *ALD5* | 272 ± 15 | 194 ± 8 | 68 ± 5 | 78 ± 18 | 175 ± 19 | 157 ± 21 |
| *ALD6* | 824 ± 36 | 554 ± 47 | 1350 ± 94 | 888 ± 161 | 1061 ± 116 | 1972 ± 194 |
| *ACS1* | 5 ± 1 | 11 ± 2 | 1271 ± 155 | 823 ± 50 | 1611 ± 391 | 2213 ± 337 |
| *ACS2* | 319 ± 13 | 285 ± 13 | 784 ± 75 | 582 ± 28 | 293 ± 18 | 312 ± 14 |
| *ACC1* | 97 ± 5 | 120 ± 5 | 164 ± 8 | 176 ± 8 | 195 ± 9 | 160 ± 7 |
| *FAS1* | 89 ± 6 | 177 ± 6 | 104 ± 4 | 117 ± 11 | 251 ± 9 | 261 ± 9 |
| *FAS2* | 125 ± 7 | 140 ± 7 | 112 ± 4 | 105 ± 7 | 143 ± 6 | 126 ± 8 |
| **Other target genes indicated by Larochelle *et al*. and Hector *et al*.** | | | | | | |
| *ILV5* | 2174 ± 85 | 1583 ± 80 | 464 ± 57 | 444 ± 152 | 1342 ± 172 | 1022 ± 63 |
| *GOR1* | 25 ± 3 | 91 ± 6 | 193 ± 12 | 205 ± 29 | 205 ± 10 | 272 ± 26 |
| *YEF1* | 12 ± 1 | 13 ± 2 | 34 ± 3 | 38 ± 4 | 235 ± 20 | 1691 ± 324 |
| *ADH6* | 232 ± 9 | 460 ± 12 | 72 ± 12 | 55 ± 28 | 116 ± 31 | 191 ± 33 |
| *IDP2* | 13 ± 2 | 19 ± 2 | 1492 ± 75 | 1085 ± 37 | 668 ± 46 | 944 ± 95 |
| YMR315W | 136 ± 5 | 475 ± 17 | 193 ± 16 | 309 ± 30 | 325 ± 16 | 1250 ± 136 |
| **Target genes indicated by Ouyang *et al*.** | | | | | | |
| *TAL1* | 949 ± 49 | 821 ± 20 | 333 ± 13 | 475 ± 32 | 961 ± 20 | 1626 ± 188 |
| *GND1* | 1033 ± 35 | 1019 ± 20 | 73 ± 4 | 204 ± 33 | 556 ± 72 | 1583 ± 216 |
| *SOL3* | 308 ± 23 | 158 ± 9 | 52 ± 5 | 36 ± 6 | 242 ± 26 | 184 ± 11 |
| *TKL1* | 603 ± 17 | 545 ± 23 | 161 ± 15 | 132 ± 59 | 266 ± 62 | 307 ± 20 |
| YMR315W | 136 ± 5 | 475 ± 17 | 193 ± 16 | 309 ± 30 | 325 ± 16 | 1250 ± 136 |
| *ALD6* | 824 ± 36 | 554 ± 47 | 1350 ± 94 | 888 ± 161 | 1061 ± 116 | 1972 ± 194 |
| *YEF1* | 12 ± 1 | 13 ± 2 | 34 ± 3 | 38 ± 4 | 235 ± 20 | 1691 ± 324 |
| *NDE1* | 106 ± 8 | 728 ± 22 | 621 ± 31 | 614 ± 30 | 461 ± 46 | 793 ± 46 |
| *GCY1* | 26 ± 3 | 257 ± 19 | 302 ± 37 | 684 ± 96 | 349 ± 33 | 767 ± 58 |
| *IFA38* | 239 ± 13 | 218 ± 12 | 127 ± 10 | 89 ± 14 | 124 ± 7 | 125 ± 5 |
| *SFC1* | 2 ± 1 | 126 ± 10 | 2705 ± 110 | 1772 ± 67 | 516 ± 27 | 1443 ± 196 |
| *GOR1* | 25 ± 3 | 91 ± 6 | 193 ± 12 | 205 ± 29 | 205 ± 10 | 272 ± 26 |
| *AGX1* | 12 ± 2 | 111 ± 10 | 1918 ± 68 | 2242 ± 219 | 534 ± 23 | 1408 ± 52 |
| *RPS30B* | 1281 ± 76 | 927 ± 62 | 337 ± 34 | 308 ± 80 | 594 ± 69 | 511 ± 41 |
| *RPS12* | 2565 ± 87 | 1516 ± 36 | 183 ± 21 | 200 ± 134 | 729 ± 118 | 585 ± 32 |
| *RPS19A* | 965 ± 47 | 490 ± 30 | 125 ± 11 | 122 ± 62 | 385 ± 52 | 280 ± 16 |
| *KNH1* | 37 ± 5 | 31 ± 3 | 63 ± 11 | 55 ± 4 | 211 ± 21 | 204 ± 19 |
| *IMA5* | 5 ± 1 | 51 ± 10 | 7 ± 1 | 100 ± 9 | 41 ± 8 | 471 ± 116 |
| *PMA1* | 1975 ± 252 | 1610 ± 65 | 618 ± 78 | 524 ± 38 | 513 ± 78 | 530 ± 46 |
| *ATR1* | 273 ± 12 | 208 ± 9 | 38 ± 3 | 54 ± 6 | 44 ± 5 | 65 ± 5 |
| YPR015C | 7 ± 2 | 17 ± 3 | 45 ± 5 | 62 ± 7 | 34 ± 6 | 60 ± 6 |
| *ADR1* | 3 ± 1 | 52 ± 3 | 95 ± 8 | 90 ± 6 | 68 ± 5 | 96 ± 4 |
| *CMR3* | 47 ± 3 | 31 ± 3 | 71 ± 6 | 53 ± 6 | 46 ± 5 | 48 ± 5 |
| *MSN4* | 56 ± 7 | 75 ± 5 | 47 ± 3 | 53 ± 7 | 46 ± 4 | 55 ± 5 |
| *RGM1* | 41 ± 6 | 28 ± 4 | 61 ± 7 | 34 ± 5 | 51 ± 9 | 33 ± 5 |
| *VHR1* | 49 ± 4 | 48 ± 2 | 67 ± 5 | 44 ± 4 | 40 ± 5 | 36 ± 3 |
| *DSD1* | 51 ± 4 | 73 ± 7 | 118 ± 7 | 82 ± 8 | 124 ± 6 | 147 ± 13 |
| YDR248C | 86 ± 6 | 154 ± 14 | 64 ± 6 | 108 ± 15 | 141 ± 10 | 215 ± 25 |
| YLR152C | 23 ± 3 | 150 ± 7 | 133 ± 13 | 116 ± 10 | 71 ± 4 | 237 ± 55 |
| *APT2* | 82 ± 5 | 122 ± 9 | 62 ± 7 | 80 ± 14 | 118 ± 14 | 130 ± 15 |
| *BMH1* | 2298 ± 230 | 2649 ± 78 | 1263 ± 69 | 1618 ± 65 | 2074 ± 223 | 2534 ± 128 |
| YMR265C | 37 ± 3 | 54 ± 5 | 84 ± 6 | 85 ± 7 | 47 ± 2 | 35 ± 2 |
| *HEM1* | 115 ± 9 | 78 ± 4 | 218 ± 7 | 134 ± 7 | 158 ± 18 | 138 ± 8 |
| *MSC6* | 64 ± 5 | 120 ± 4 | 105 ± 6 | 86 ± 8 | 59 ± 6 | 46 ± 4 |
| *GGC1* | 370 ± 23 | 612 ± 58 | 347 ± 15 | 239 ± 47 | 184 ± 15 | 283 ± 21 |
| *DRE2* | 248 ± 8 | 145 ± 9 | 143 ± 8 | 164 ± 10 | 152 ± 16 | 122 ± 20 |

Hector RE, Bowman MJ, Skory CD *et al.* The *Saccharomyces cerevisiae* YMR315W gene encodes an NADP(H)-specific oxidoreductase regulated by the transcription factor Stb5p in response to NADPH limitation. *New Biotechnol* 2009;**26**:171-80.

Larochelle M, Drouin S, Robert F *et al.* Oxidative stress-activated zinc cluster protein Stb5 has dual activator/repressor functions required for pentose phosphate pathway regulation and NADPH production. *Mol Cell Biol* 2006;**26**:6690-701.

Ouyang L, Holland P, Lu H *et al.* Integrated analysis of the yeast NADPH-regulator Stb5 reveals distinct differences in NADPH requirements and regulation in different states of yeast metabolism. *FEMS Yeast Res* 2018;**18**:foy091.
